# The Dietary Fiber Inulin Slows Progression of Chronic Kidney Disease–Mineral Bone Disorder (CKD‐MBD) in a Rat Model of CKD

**DOI:** 10.1002/jbm4.10837

**Published:** 2023-12-07

**Authors:** Annabel Biruete, Neal X. Chen, Corinne E. Metzger, Shruthi Srinivasan, Kalisha O'Neill, Paul B. Fallen, Austin Fonseca, Hannah E. Wilson, Henriette de Loor, Pieter Evenepoel, Kelly S. Swanson, Matthew R. Allen, Sharon M. Moe

**Affiliations:** ^1^ Department of Nutrition Science Purdue University West Lafayette IN USA; ^2^ Department of Medicine, Division of Nephrology Indiana University School of Medicine Indianapolis IN USA; ^3^ Department of Anatomy, Cell Biology, and Physiology Indiana University School of Medicine Indianapolis IN USA; ^4^ KU Leuven Department of Microbiology and Immunology Nephrology and Renal Transplantation Research Group, KU Leuven Leuven Belgium; ^5^ Department of Nephrology and Renal Transplantation University Hospitals Leuven Leuven Belgium; ^6^ Department of Animal Sciences University of Illinois at Urbana‐Champaign Urbana IL USA

**Keywords:** BONE, CARDIOVASCULAR, CKD‐MINERAL BONE DISORDER, DIETARY FIBER, INULIN

## Abstract

Chronic kidney disease (CKD)–mineral bone disorder (CKD‐MBD) leads to fractures and cardiovascular disease. Observational studies suggest beneficial effects of dietary fiber on both bone and cardiovascular outcomes, but the effect of fiber on CKD‐MBD is unknown. To determine the effect of fiber on CKD‐MBD, we fed the Cy/+ rat with progressive CKD a casein‐based diet of 0.7% phosphate with 10% inulin (fermentable fiber) or cellulose (non‐fermentable fiber) from 22 weeks to either 30 or 32 weeks of age (~30% and ~15% of normal kidney function; CKD 4 and 5). We assessed CKD‐MBD end points of biochemistry, bone quantity and quality, cardiovascular health, and cecal microbiota and serum gut‐derived uremic toxins. Results were analyzed by two‐way analysis of variance (ANOVA) to evaluate the main effects of CKD stage and inulin, and their interaction. The results showed that in CKD animals, inulin did not alter kidney function but reduced the increase from stage 4 to 5 in serum levels of phosphate and parathyroid hormone, but not fibroblast growth factor‐23 (FGF23). Bone turnover and cortical bone parameters were similarly improved but mechanical properties were not altered. Inulin slowed progression of aorta and cardiac calcification, left ventricular mass index, and fibrosis. To understand the mechanism, we assessed intestinal microbiota and found changes in alpha and beta diversity and significant changes in several taxa with inulin, together with a reduction in circulating gut derived uremic toxins such as indoxyl sulfate and short‐chain fatty acids. In conclusion, the addition of the fermentable fiber inulin to the diet of CKD rats led to a slowed progression of CKD‐MBD without affecting kidney function, likely mediated by changes in the gut microbiota composition and lowered gut‐derived uremic toxins. © 2023 The Authors. *JBMR Plus* published by Wiley Periodicals LLC. on behalf of American Society for Bone and Mineral Research.

## Graphical abstract

Biochemistries of CKD‐MBD progressively increased with advancing CKD with variable effect of dietary inulin. Serum/plasma was collected at the time of euthanasia and analyzed by either colorimetric assays or ELISA. Two‐way ANOVA compared severity of CKD/age (stage 4 vs. 5) and fiber (10% inulin in the diet (gray bar) compared to cellulose (black bar) in the diet). The mean value from the normal animals from both time points are shown as the dashed black line and was not included in the statistical model. The *p* values for age, inulin, and an interaction of age and inulin are shown in each graph. All measures worsened from stage 4 to stage 5 except 8‐OHdG, a measure of oxidative stress. At stage 5, post hoc testing showed that inulin significantly lowered phosphorus, PTH, and 8‐OHdG (all *p* < 0.01). At stage 4 CKD, inulin increased iFGF23 (*p* = 0.048), but not at stage 5. *n* = 10 to 12 for each group shown as individual symbols.

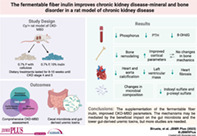

## Introduction

Chronic kidney disease–mineral bone disorder (CKD‐MBD) is a triad of abnormal biochemistries, impaired bone health (renal osteodystrophy), and extraosseous calcification that results in increased fractures and cardiovascular disease.^[^ ^1^ ^]^ Bone fractures are progressively increased at every stage of CKD compared to a similarly aged general population,^[^ ^2^ ^]^ and mortality after a hip fracture is doubled in patients with CKD.^[^ ^3^ ^]^ However, neither bone density nor bone volume fully accounts for the high fracture incidence, suggesting CKD specific alterations.^[^ ^4^ ^]^ Similarly, cardiovascular events and mortality are increased in CKD and traditional risk factors and laboratory‐based diagnostic cardiovascular biomarkers do not fully explain or predict the increased risk,^[^ ^5^ ^]^ supporting the importance of nontraditional risk factors specific to CKD. One group of risk factors are uremic toxins, defined as substances elevated in CKD, associated with disease in patients, and toxicity in vitro.^[^ ^6^ ^]^ Uremic toxins include gut‐derived toxins such as indoxyl sulfate, but also CKD‐MBD toxins such as elevated phosphate, parathyroid hormone (PTH), and fibroblast growth factor‐23 (FGF23) that have been linked with arterial calcification, left ventricular hypertrophy (LVH), and myocardial fibrosis in animal models and patients with CKD.^[^ ^7^, ^8^, ^9^, ^10^ ^]^

Fermentable fiber, including inulin, can alter intraluminal pH, improve mineral solubility, and enhance mineral absorption,^[^ ^11^ ^]^ potentially impacting CKD‐MBD. Fermentable fiber is known to affect the gut microbiome in CKD,^[^ ^12^, ^13^ ^]^ and the gut microbiota have been shown to modulate bone response to PTH in rodent models.^[^ ^14^, ^15^ ^]^ In CKD patients, alterations in the microbiome may decrease the serum levels of the gut‐derived uremic toxins indoxyl sulfate and p‐cresyl sulfate in CKD and hemodialysis patients.^[^ ^12^, ^16^ ^]^ In the general population, increased fiber intake improved bone mass,^[^ ^17^ ^]^ reduced fractures,^[^ ^18^ ^]^ and reduced cardiovascular events.^[^ ^19^ ^]^ The goal of this study was to test the hypothesis that the fermentable fiber inulin will improve CKD‐MBD in our established slowly progressive CKD rat model.

## Materials and Methods

### Experimental design

The Cy/+_IU_ colony of rats is bred at Indiana University: Cy/+ rats have progressive kidney disease due to cyst growth although the defect is not orthologous to human polycystic kidney disease with gene defects in ciliary proteins.^[^ ^20^, ^21^ ^]^ The Cy/+ rat model develops CKD‐MBD spontaneously on a normal phosphate diet, with a much faster progression (30 vs. 80 weeks) to end‐stage disease in male animals versus females even with ovariectomy^[^ ^22^, ^23^ ^]^ and, therefore, only males were used. Male Cy/+_IU_ rats (CKD hereafter) and their normal littermates (NL) were fed the autoclaved grain‐based diet (Teklad 2018SX; Teklad, Indianapolis, IN, USA) until 22 weeks of age and then switched to a casein‐based diet (TD.04539; 18% casein protein, 0.7% Pi, 0.6% Ca; Teklad) with cellulose, a minimally fermented fiber. Treatment with inulin began at 22 weeks of age (~50% normal glomerular filtration rate [GFR]). Two concurrent studies (30‐week and 32‐week duration studies) were conducted, hereafter referred to CKD stage 4 and 5. Similar to observations in humans,^[^ ^24^ ^]^ we see a dramatic increase in PTH, FGF23, and phosphate with marked cortical bone changes and the development of arterial calcification between, stage 4 to 5 CKD.^[^ ^25^ ^]^ Three groups of animals were compared (*n* = 12–14/group based on power required due to PTH variability) in each study: (i) Normal littermate animals (NL) with casein/cellulose diet; (ii) CKD animals with casein/cellulose; and (iii) CKD animals with 10% inulin (CKD/inulin) instead of cellulose. Calcein (30 mg/kg) was injected 14 and 4 days prior to euthanasia, at which time tissue and cecal digesta were collected, weighed, and stored for analyses. The Indiana Institutional Animal Care and Use Committee approved all procedures.

### Blood measures

Plasma was analyzed for blood urea nitrogen, creatinine, calcium, and phosphorus using colorimetric assays (Pointe Scientific, Canton, MI, USA, or BioAssay Systems, Hayward, CA, USA). Plasma intact PTH, serum C‐terminal and intact FGF23, and oxidative stress marker 8‐hydroxy‐2′‐deoxyguanosine (8‐OHdG) were determined by enzyme‐linked immunosorbent assay (ELISA) (Quidel, San Diego, CA, USA, or Enzo Life Sciences, Farmingdale, NY, USA).^[^ ^26^ ^]^ Serum uremic toxins were measured by ultra‐performance liquid chromatography–tandem mass spectrometry.^[^ ^27^ ^]^

### Bone measures

Micro–computed tomography (μCT) was performed on the proximal tibia using a Skyscan 1172 (Bruker, Billerica, MA, USA) at 12‐μm resolution as published.^[^ ^25^ ^]^ Briefly, a 1‐mm region of interest starting roughly 0.5 mm from the distal end of the growth plate was used for analysis of trabecular bone. Cortical bone was assessed as the average of five slices 4 mm distal to the trabecular region. All CT analyses were done in accordance with standard guidelines.^[^ ^28^ ^]^ For dynamic bone histomorphometry, unmineralized proximal tibia were fixed in neutral buffered formalin then subjected to serial dehydration and embedded in methyl methacrylate (Sigma Aldrich, St. Louis, MO, USA). Serial frontal sections were cut 4 μm thick and left unstained for analysis of fluorochrome calcein labels.

Histomorphometric analyses were performed using BIOQUANT Image Analysis (Nashville, TN, USA). A standard region of interest of trabecular bone excluding primary spongiosa and endocortical surfaces was assessed at magnification ×20 and utilized measures and nomenclature following published standards.^[^ ^29^ ^]^ Additional 4‐μm sections were stained for tartrate‐resistant alkaline phosphatase (TRAP) and counterstained with toluidine blue for analysis of osteoclast‐covered trabecular surfaces normalized to total trabecular bone surface.

Whole femurs frozen in phosphate‐buffered saline were μCT scanned using a Skyscan 1176 (Bruker, Billerica, MA, USA) at 18‐μm resolution to assess geometric properties at the mid‐diaphysis. Femoral diaphysis mechanical properties were assessed in three‐point bending using standard instrumentation (Test Resources, Shakopee, MN, USA). Bones were thawed to room temperature, kept hydrated in saline, and placed posterior surface down on bottom supports (span = 18 mm). The upper support was brought down in contact with the specimen's anterior surface and testing was conducted at a displacement rate of 2 mm/min with a 667.2‐N load cell. Force versus displacement data were collected at 10 Hz and structural parameters were determined from curves using a customized MATLAB program (MathWorks, Natick, MA, USA). Material properties were estimated using standard beam‐bending equations.^[^ ^30^ ^]^

### Aortic arch and heart calcification and left ventricular mass index

Segments of the aortic arch and heart were separately washed in 0.9% saline and incubated in 0.6 N HCl for 48 h. The supernatant was analyzed for calcium using the *o*‐cresolphthalein complex one method (Calcium kit; Pointe Scientific) normalized by tissue dry weight.^[^ ^31^, ^32^ ^]^ Left ventricular mass index (LVMI) was determined by dividing total heart weight by body weight. Heart tissue messenger RNA (mRNA) expression of transforming growth factor‐β (TGF‐β) was determined by real‐time quantitative polymerase chain reaction (qPCR) with primer Rn00572010_m1.

### Heart histologic analysis

The hearts were sectioned just below the valves and fixed in 10% (vol/vol) neutral buffered formalin, paraffin embedded, and stained for fibrosis by Masson's Trichrome stain and calcification by Von Kossa with fast green as published.^[^ ^33^ ^]^ Bright‐field mosaic images of the entire tissue sections were acquired with a Keyence BZ‐X810 microscope using a Nikon PlanFluor 10×/0.3 objective lens.

### Gut microbiota of cecal digesta

Rats are cecal fermenters, and therefore total genomic DNA from cecal digesta (proximal large intestine) were extracted (Qiagen, Ann Arbor, MI, USA), double‐stranded DNA was quantified using the Clariostar spectrometer (BMG Labtech, Cary, NC, USA), and quality assessed by electrophoresis with 2% Agarose EX‐gels using the E‐Gel iBase (Invitrogen, Grand Island, NY, USA). Fluidigm Access Array (UC Davis, Davis, CA, USA) was used to generate 16S rRNA gene amplicons, in combination with Roche High Fidelity Fast Start Kit (Roche Diagnostics, Mannheim, Germany). Primers 515F and 806R targeting a 252‐basepair (bp) fragment of the V4 region of the bacterial 16S rRNA were amplified, sequenced, and analyzed as in the Supplementary Methods.

### Intestinal phosphate transporters, RNA isolation, and real‐time PCR

Intestinal tissues were flushed with 0.9% saline using a gavage needle to remove luminal contents, cut transversally with scissors, the mucosa scraped with microscope cover slip, and the tissue placed in a microcentrifuge tube and flash‐frozen. Duodenum was considered 1 cm proximal to the pylorus until the suspensory muscle of the duodenum; ileum was considered 30 cm proximal to the ileocecal valve, and jejunum the rest of the tissue. Cecum RNA was extracted from whole tissue instead of the mucosa scraping. Real‐time PCR conducted with target specific PCR primers (ThermoFisher Life Technologies as described.^[^ ^34^, ^35^ ^]^) The delta‐delta‐comparative threshold (ΔΔCT) method was used to analyze the relative change in gene expression normalized to *Rplp0*.

### Statistics

The question of interest was a comparison between CKD with and without inulin. We have previously shown dramatic differences between CKD and NL littermates, and therefore the NL rat data was used to determine if inulin normalized findings and not included in the statistical analyses (but represented by the black dashed line in all figures). Statistical analyses were conducted by first excluding outliers using ROUT (*Q* = 1%), followed by a normality test (*p* < 0.05 with Anderson‐Darling Test). Data was log‐transformed if non‐normal before analyses. For comparisons between the two studies (30‐week end point/stage 4 and 32‐week end point/stage 5) we used a two‐way ANOVA for CKD severity (stage 4 vs. 5) and fiber treatment (inulin vs. cellulose) and followed by Tukey's multiple comparisons test; referred to as “post‐hoc testing” in the results section. For end points only assessed at stage 5 CKD (intestinal phosphate transporter gene expression, bone mechanical test and heart TGF‐β expression) we used a one‐way ANOVA and, if overall ANOVA showed *p* < 0.05, we conducted within group comparisons by Dunnett's post hoc analyses, comparing untreated CKD rats versus each of the other groups. The results are expressed as means ± standard deviation (SD) with *p* < 0.05 considered significant (GraphPad Prism Software version 9.4.1; GraphPad, La Jolla, CA, USA).

## Results

### Dietary inulin improved CKD‐MBD biochemistries

**Fig. 1:**
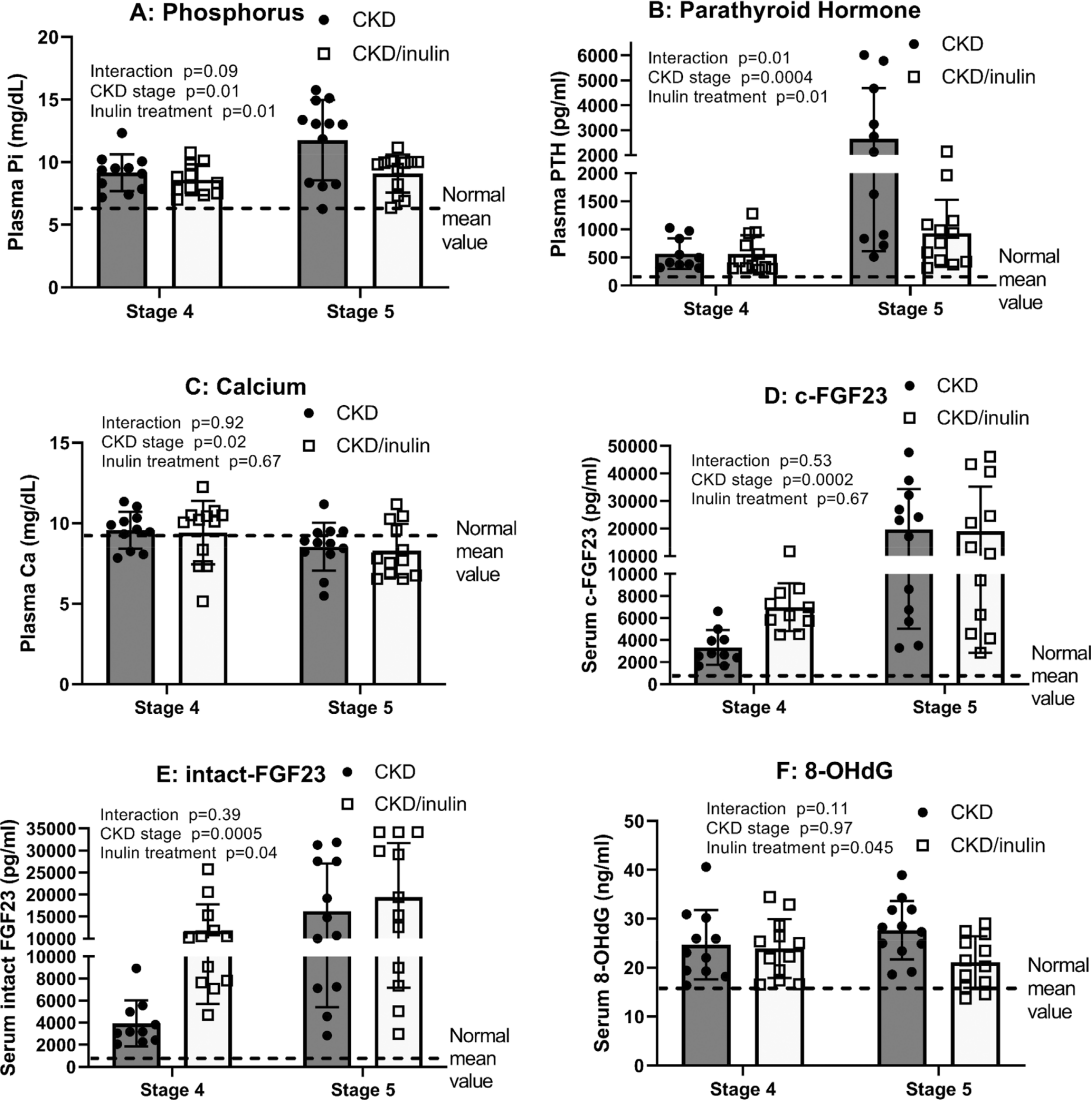
Biochemistries of CKD‐MBD progressively increased with advancing CKD with variable effect of dietary inulin. Serum/plasma was collected at the time of euthanasia and analyzed by either colorimetric assays or ELISA. Two‐way ANOVA compared severity of CKD/age (stage 4 vs. 5) and fiber (10% inulin in the diet (gray bar) compared to cellulose (black bar) in the diet). The mean value from the normal animals are shown as the dashed black line and was not included in the statistical model. The *p* values for age, inulin, and an interaction of age and inulin are shown in each graph. All measures worsened from stage 4 to stage 5 except 8‐OHdG, a measure of oxidative stress. At stage 5, post hoc testing showed that inulin significantly lowered phosphorus, PTH and 8‐OHdG (all *p* < 0.01). At stage 4 CKD, inulin increased iFGF23 (*p* = 0.048), but not at stage 5. *n* = 10 to 12 for each group shown as individual symbols.

CKD rats had a progressive decline in kidney function (elevated blood urea nitrogen [BUN], creatinine) and increase in kidney weight due to cyst growth from stage 4 to 5, both different than NL animals but unaffected by inulin (Table S1). The plasma phosphorus (Fig. 1A, *p* = 0.01) and PTH (Fig. 1*B*, *p* = 0.0004) levels increased in CKD rats from stage 4 to 5. Inulin treatment significantly decreased both plasma phosphorus and PTH levels, effectively slowing progression of CKD‐MBD (Fig. 1A,B; post hoc PTH at CKD stage 5 *p* = 0.0004). There was a decrease in calcium levels from stage 4 to 5, but no effect of inulin (Fig. 1C). Both c‐terminal FGF23 and intact FGF23 increased from stage 4 to 5, with an effect of inulin to increase intact FGF23 at stage 4 (Fig. 1D,E). Oxidative stress as measured by 8‐OHdG, a marker of DNA oxidation, did not increase over time, with only a modest reduction by inulin (*p* = 0.04; Fig. 1F), driven by stage 5 results. Overall, these results demonstrated that inulin did not alter CKD progression but reduced plasma phosphate and PTH.

### The effect of inulin treatment on bone in CKD rats

**Fig. 2:**
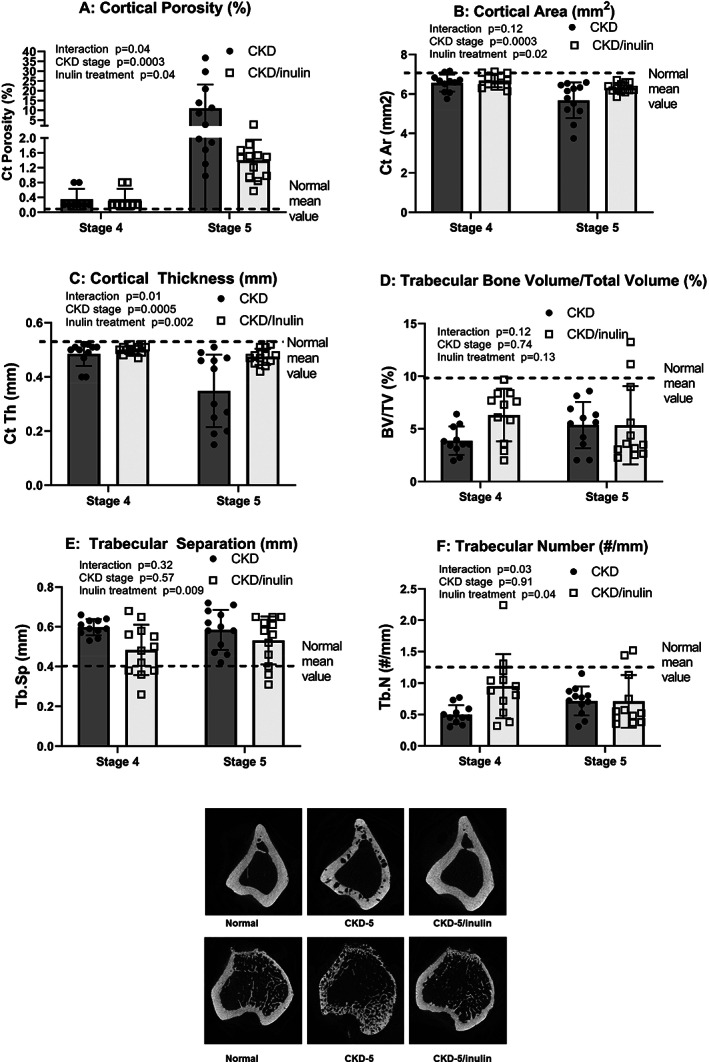
Inulin treatment improved cortical bone changes assessed by μCT, with some effects on trabecular bone. Bones were collected at the time of euthanasia and analyzed by μCT. Two‐way ANOVA compared severity of CKD (stage 4 and 5) and with fiber (10% inulin in the diet (gray bar) compared to cellulose (black bar) in the diet). The mean value from the normal animals are shown as the dashed black line and was not included in the statistical model. The *p* values for CKD stage, inulin, and an interaction of stage and inulin are shown in each graph. Cortical porosity (*A*) increased with CKD stage, and was improved with inulin in the diet and interaction between these. Cortical area (*B*) and thickness (*C*) decreased from stage 4 to 5 and was improved with inulin treatment, but only significant at stage 5 by post hoc testing (*p* < 0.007 for both measures). In contrast, there was no CKD stage effect or inulin effect on trabecular volume/total volume (*D*). There was no CKD stage effect on trabecular separation and number (*E*,*F*), but inulin decreased trabecular separation and increased trabecular number (*p* = 0.009 and 0.04, respectively). *n* = 10–12 for each group shown as individual symbols. Representative images from the μCT (*G*): The left panel is from a NL animal, the middle panel from CKD stage 5 animal (taken from an animal with average value for % cortical porosity), and the right panel from CKD stage 5 animal treated with inulin demonstrating a marked reduction in porosity.

μCT analysis of cortical bone at the proximal tibia showed cortical porosity dramatically increased from stage 4 to 5 in CKD rats (Fig. 2A,G; *p* = 0.01) and was reduced by inulin (*p* = 0.035; effect only at stage 5 (*p* = 0.001)). Cortical area and thickness (Fig. 2B,C) decreased from stage 4 to 5 (*p* = 0.0003, *p* = 0.0005, respectively) and both improved with inulin treatment (*p* = 0.023, *p* = 0.002, respectively), especially at CKD stage 5 (both *p* < 0.007 by post hoc testing). In contrast, there was no CKD stage effect or inulin effect on trabecular bone volume/total volume by microCT (Fig. 2D), but inulin decreased trabecular separation and increased trabecular number (*p* = 0.009 and 0.04, respectively, Fig. 2E,F). Representative images of bones from NL, CKD, and CKD fed inulin are shown in Fig. 2G.

**Fig. 3:**
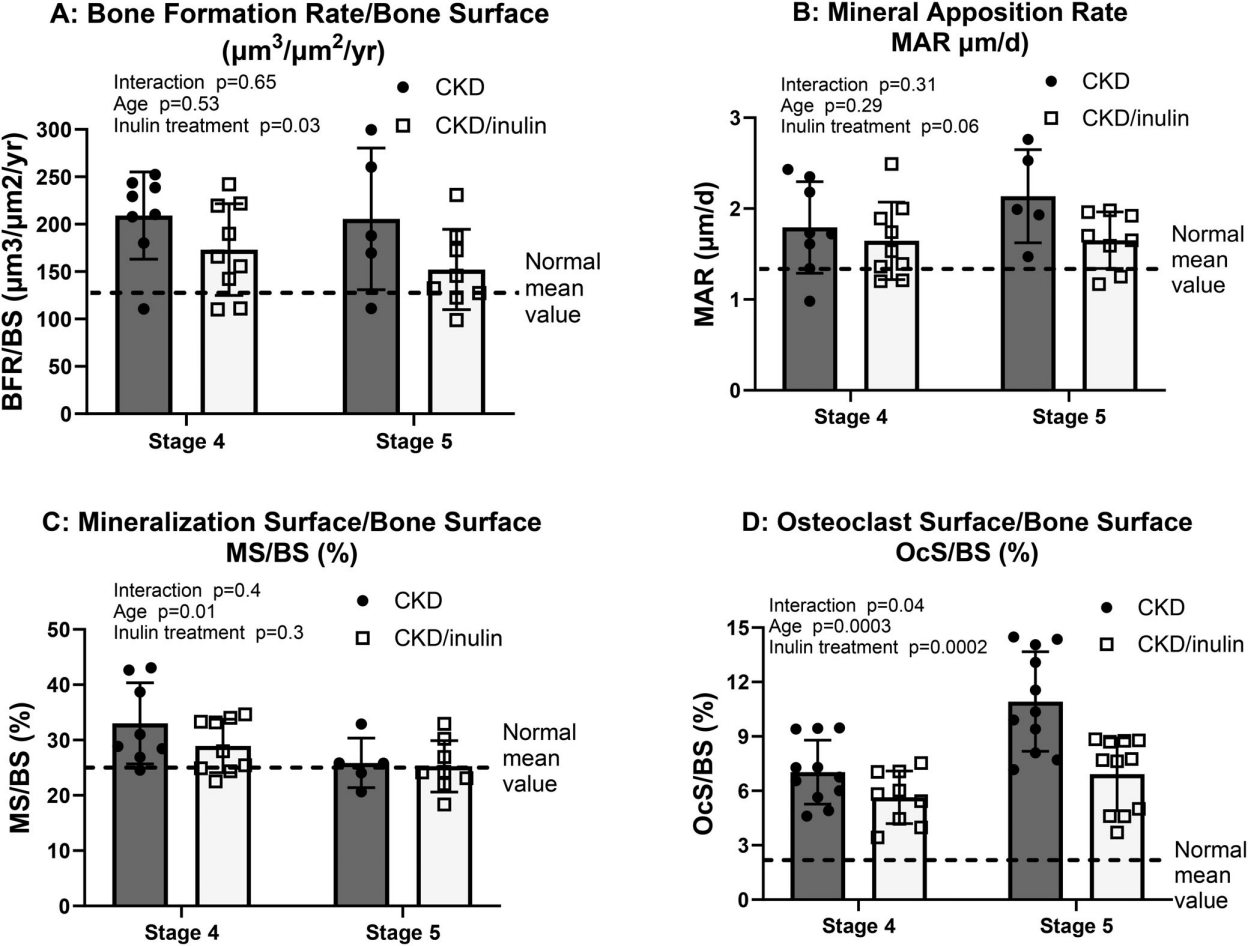
Inulin decreased CKD‐induced high bone turnover. Bones were collected at the time of euthanasia and embedded for histomorphometry. Two‐way ANOVA compared severity of CKD (stage 4 vs. 5) and fiber (10% inulin in the diet (gray bar) compared to cellulose (black bar) in the diet). The mean value from the normal animals are shown as the dashed black line and was not included in the statistical model. The *p* values for stage, inulin, and an interaction of CKD stage and inulin are shown in each graph. Bone formation rate (BFR/BS; *A*) and mineral apposition rate (MAR; *B*) in CKD rats did not increase with advancing CKD; inulin treatment decreased bone formation rate (*p* = 0.028) with a similar trend for MAR (*p* = 0.06). In contrast, mineralizing surface (MS/BS; *C*) decreased with CKD stage 5 (*p* = 0.015) but was unaffected by inulin. Bone tartrate resistant alkaline phosphatase (TRAP) staining to quantify osteoclast surface (OcS/BS; *D*) progressively increased from stage 4 to 5 CKD (*p* = 0.0002). Inulin decreased osteoclast surface (*p* = 0.0002), with post hoc comparison only different at stage 5 CKD (*p* < 0.0001). *n* = 5–12 for each group shown as individual symbols.

Dynamic histomorphometry demonstrated higher trabecular bone formation rate (BFR/BS) and mineral apposition rate (MAR) in CKD rats at both stage 4 to 5 compared to normal (Fig. 3A,B), but no increase with CKD stage. Inulin treatment decreased bone formation rate (*p* = 0.028) with a similar nonsignificant trend for MAR (*p* = 0.06). In contrast, there was decreased mineralizing surface (MS/BS) with age (*p* = 0.015) but no effect of inulin (Fig. 3C). Trabecular bone osteoclast surface (OcS/BS; Fig. 3D) progressively increased from stage 4 to 5 (*p* = 0.0002). Inulin decreased osteoclast surface (*p* = 0.0002, driven by stage 5 *p* < 0.0001 on post hoc testing).

Mechanical properties of the femur analyzed only at stage 5, demonstrated clear differences between CKD and NL animals for ultimate load, post‐yield displacement, stiffness, total work, ultimate stress, and total toughness (all *p* < 0.01), but no effect of inulin treatment on any parameter (Fig. S1).

### Dietary inulin treatment improved cardiovascular parameters in CKD rats

**Fig. 4:**
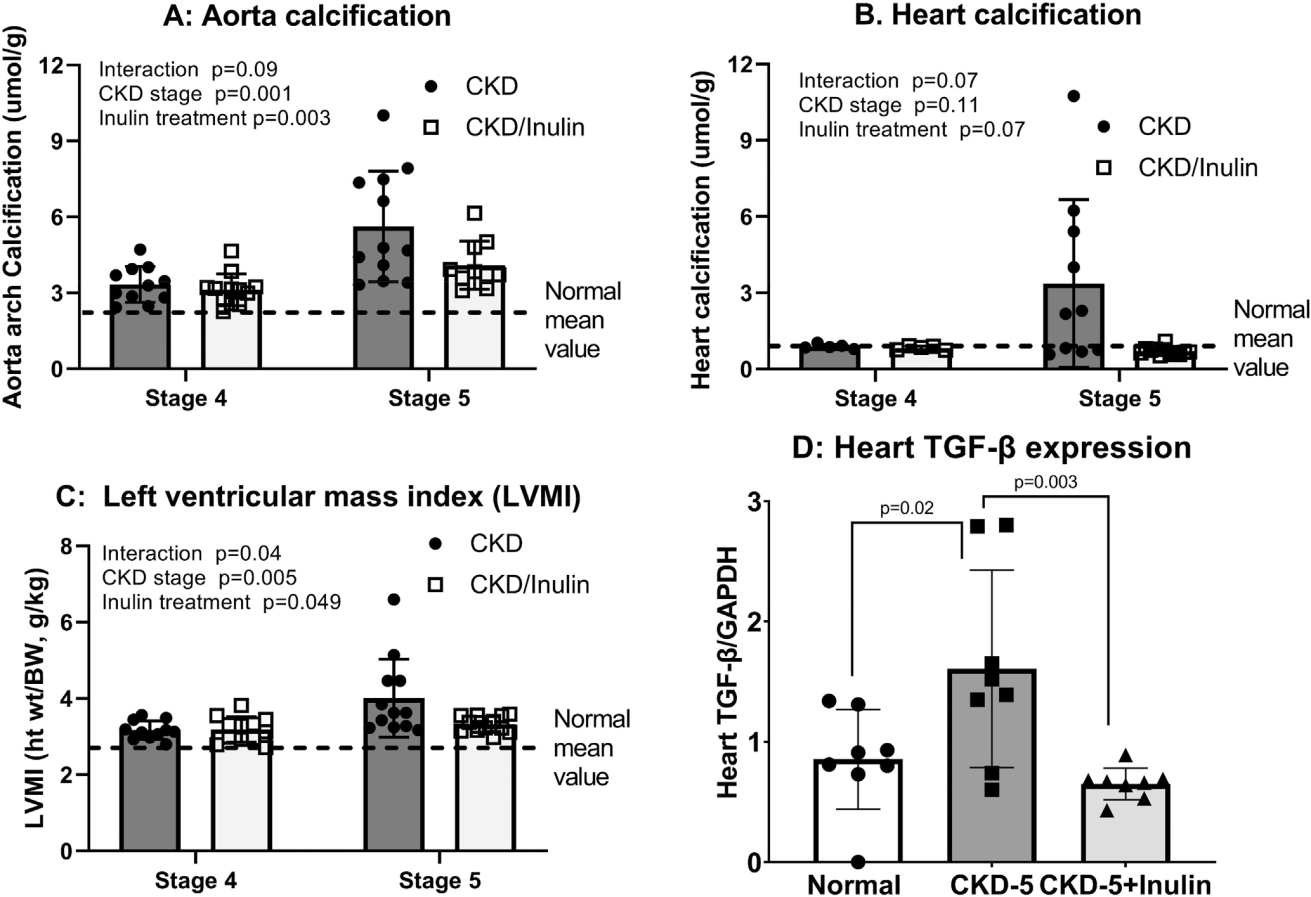
Inulin reduced aorta and cardiac calcification and decreased transforming growth factor‐β expression: The heart was collected after euthanasia, weighed to calculate left ventricular mass index (LVMI = heart weight/body weight; *C*) and then the ascending aorta and part of the ventricle collected for the quantification of calcification (*A*,*B*). Two‐way ANOVA compared severity of CKD (stage 4 vs. stage 5) and fiber (10% inulin in the diet [gray bar] compared to cellulose [black bar] in the diet). The mean value from the normal animals from both time points are shown as the dashed black line and was not included in the statistical model. The *p* values for age, inulin, and an interaction of age and inulin are shown in each graph. Aorta calcification increased from stage 4 to stage 5, and was decreased by inulin at stage 5 CKD by post hoc testing (*p* = 0.009; *A*). There was an overall trend toward an effect of inulin (*p* = 0.07, *B*) with heart calcification, but only significant at stage 5 CKD. Left ventricular mass index (*C*) increased from stage 4 to stage 5, with a trend toward a reduction by inulin (overall *p* = 0.049) but only at stage 5 (*p* = 0.01 by post hoc testing). With these latter changes, we investigated the mRNA expression of TGF‐β in the left ventricle at stage 5 CKD, and observed an increase with CKD compared to NL and a reduction with inulin (*D*). *n* = 7 to 10 for each group shown as individual symbols.

**Fig. 5:**
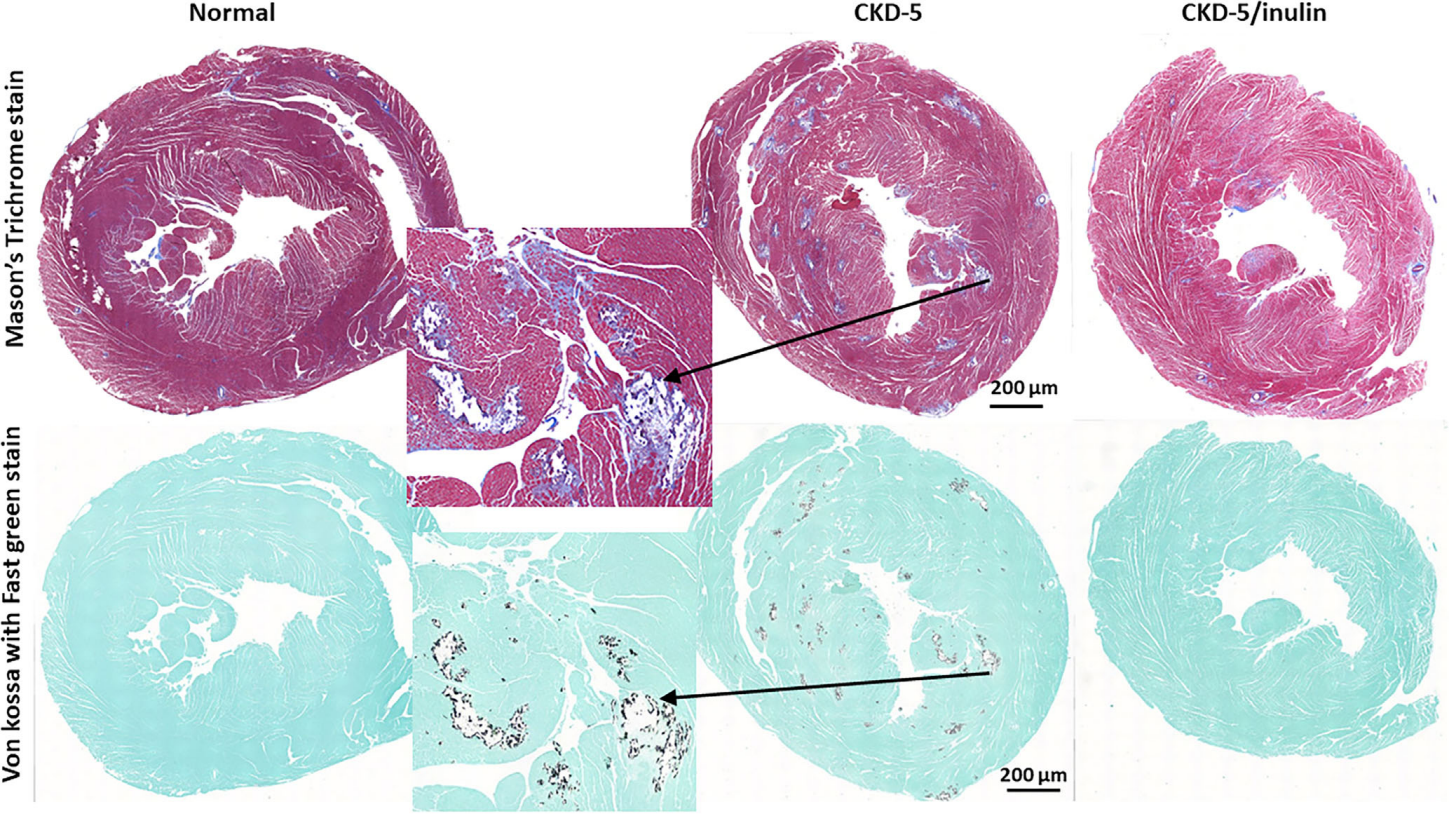
Cardiovascular calcification is improved by inulin. Representative cross sections taken just below the valves stained for fibrosis with Mason's Trichrome stain (top panel) and calcification by Von Kossa with fast green background staining (bottom panel). The CKD animals (middle panels) had increased fibrosis demonstrated by Mason's Trichrome stain (top insert blue staining) located primarily around the arterioles that were calcified (bottom insert black staining). In contrast, the fibrosis and calcification were reduced in animals given dietary inulin (far right set of panels). These data corroborate the quantification of calcium and TGFβ expression in Fig. 4. The line represents 200 μm.

We have previously demonstrated spontaneous cardiac and vascular calcification in our CKD model at older ages,^[^ ^36^ ^]^ both of which contribute to LVH and arrhythmias.^[^ ^37^ ^]^ We replicated these findings in the current study, demonstrating increased aorta calcification with progression from stage 4 to 5 (*p* = 0.001; Fig. 4A) and a reduction by inulin overall (*p* = 0.003). Cardiac calcification did not significantly increase from stage 4 to 5 (*p* = 0.11), with a trend toward an effect of inulin overall (*p* = 0.07; Fig. 4B); post hoc testing showing significance at CKD stage 5 (*p* = 0.003). Left ventricular mass index (LVMI) increased from stage 4 to 5 (*p* = 0.005) and decreased with inulin (*p* = 0.049; Fig. 4C). We therefore assessed the heart messenger RNA (mRNA) expression of TGF‐β at stage 5 and found increased expression in the CKD animals with a reduction by inulin (Fig. 4D). Qualitative histological evaluation showed the calcification was primarily in the arterioles with surrounding fibrosis (Fig. 5).

### Dietary inulin lowered the concentration of gut‐derived uremic toxins

**Fig. 6:**
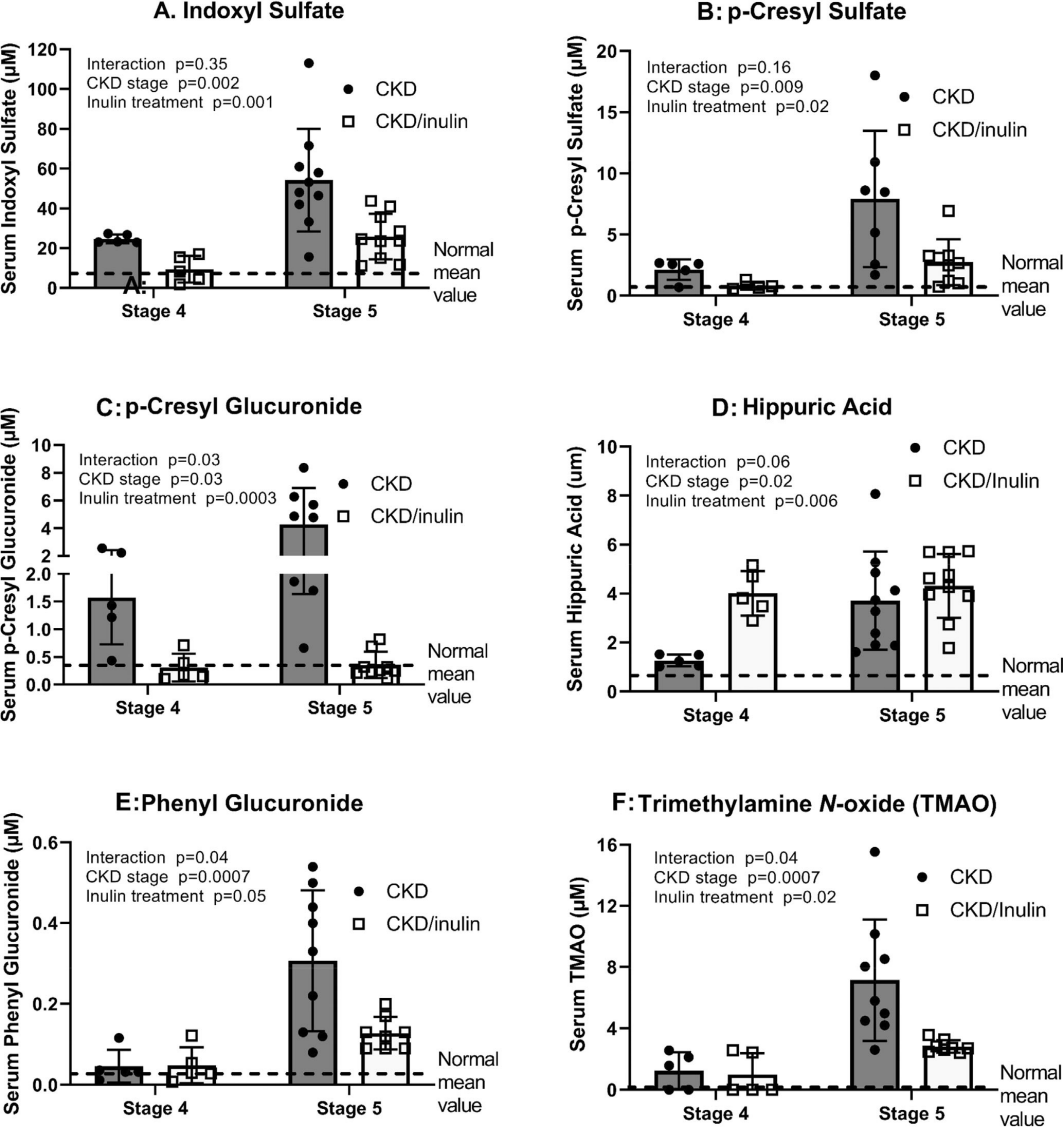
Serum uremic toxin levels are progressively increased with advancing CKD and improved with dietary inulin. Serum was collected at the time of euthanasia and analyzed by mass spectroscopy. Two‐way ANOVA compared severity of CKD (stage 4 or 5) and fiber (10% inulin in the diet (gray bar) compared to cellulose (black bar) in the diet). The mean value from the normal animals are shown as the dashed black line and was not included in the statistical model. The *p* values for stage, inulin, and an interaction of age and inulin are shown in each graph. All uremic toxin levels increased from stage 4 to 5. There was an overall effect of inulin for all the toxins, but by post hoc testing the decreases were only at stage 5 (all *p* < 0.002). The exception was hippuric acid which showed an increase by inulin at stage 4 (*p* = 0.006) and no effect at stage 5. *n* = 5 to 10 for each group shown as individual symbols.

To examine the mechanism by which inulin may alter CKD‐MBD, we first examined the RNA expression of intestinal phosphate transporters finding no effect of inulin (Fig. S2). In contrast, the serum levels of the uremic toxins indoxyl sulfate, p‐cresyl sulfate, p‐cresyl glucuronide, phenyl glucuronide, trimethylamine N‐oxide (TMAO), and hippuric acid increased from CKD stage 4 to 5 (Fig. 6, all *p* < 0.03) with an interaction of CKD stage and inulin. Post‐hoc analyses showing inulin lowered levels only at CKD stage 5 (all *p* < 0.002). In contrast inulin increased hippuric acid, driven by changes at CKD stage 4. Other measured amino acids and their metabolites were not affected by inulin (Fig. S3).

### Dietary inulin altered the cecal microbiota

**Fig. 7:**
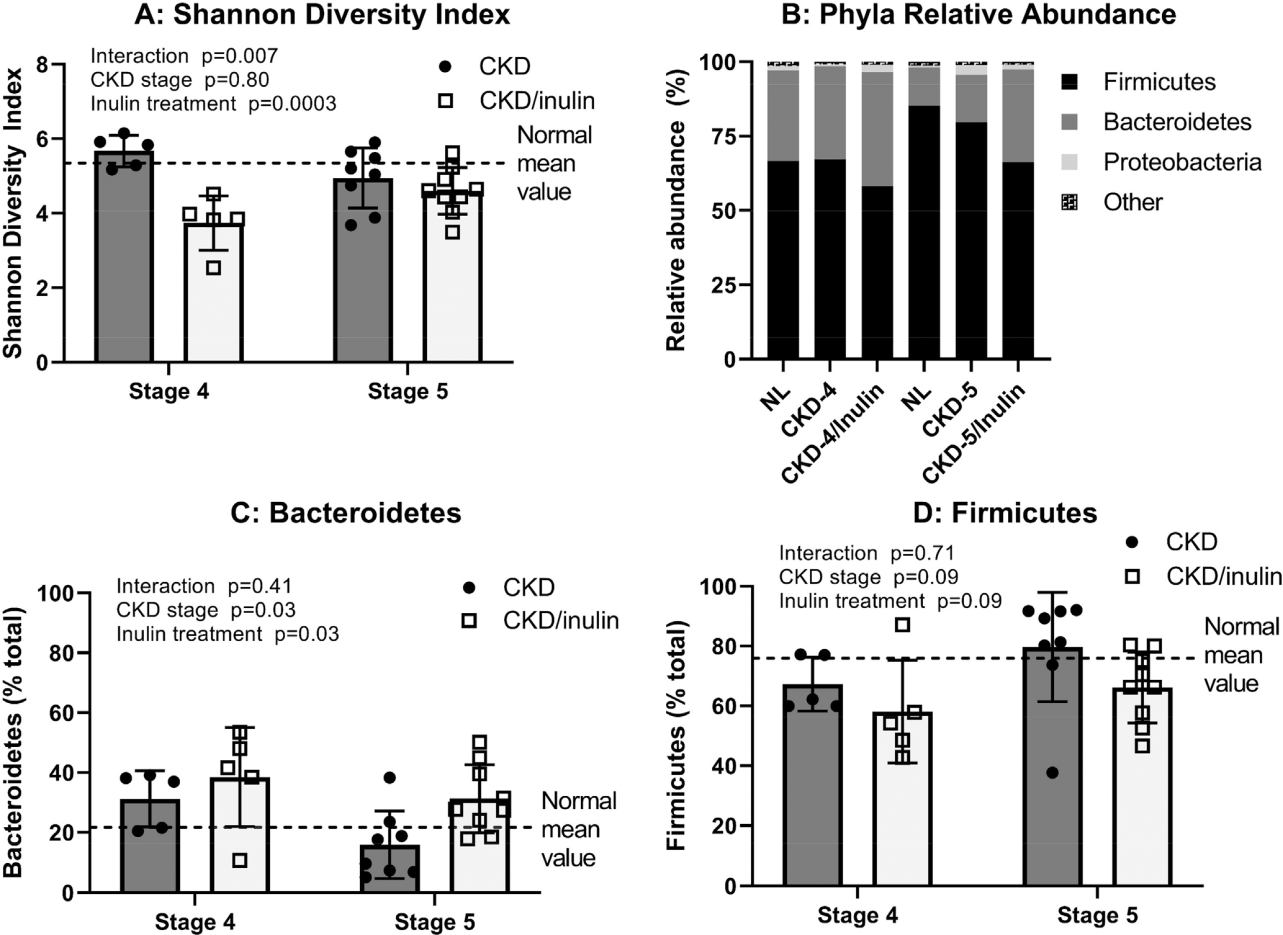
Inulin altered the cecal microbiota. Cecal genomic DNA was extracted and the V4 region of the 16S ribosomal RNA (rRNA) gene was sequenced and analyzed at stage 4 and stage 5 CKD. Two‐way ANOVA compared severity of CKD stage and fiber (10% inulin in the diet (gray bar) compared to cellulose (black bar) in the diet). The mean value from the normal animals are shown as the dashed black line and was not included in the statistical model. The *p* values for stage, inulin, and an interaction of age and inulin are shown in each graph. (*A*) Shows that the Shannon diversity index, a metric of a‐diversity, was lower with inulin at stage 4 (post hoc *p* = 0.0008), but there was no difference at stage 5. (*B*) Shows the average relative abundance at the phyla‐level in each group, where the major phyla were Firmicutes and Bacteroidetes accounting for over 95% of the relative abundance in all groups. (*C*,*D*) Show a main effect of inulin increasing the relative abundance of Bacteroidetes and a trend towards decreasing Firmicutes.

NL and CKD rats had similar a‐diversity metrics (Shannon index [Fig. 7A], ASVs, and Faith's phylogenetic diversity [Fig. S4]). CKD rats fed inulin had lower a‐diversity at stage 4 (*p* < 0.0008), but similar to NL and CKD stage 5 (*p* > 0.72 Fig. 7A). β‐diversity, or diversity between samples, using unweighted (presence vs. absence) and weighted (taking abundance into consideration) UniFrac distances also showed that the overall microbial composition was similar between NL and CKD at stages 4 and 5 (PERMANOVA q > 0.05), but CKD rats fed inulin was different from both at both stages (PERMANOVA q < 0.01, Fig. S4).

**Fig. 8:**
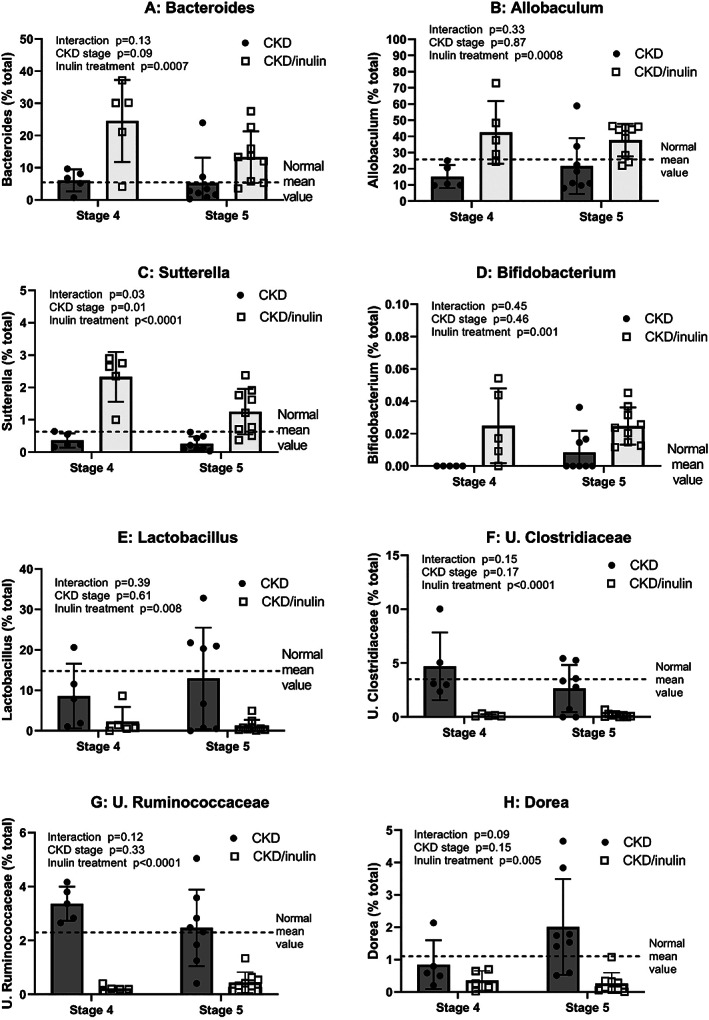
Inulin treatment led to taxonomical changes in the cecal microbiota. Cecal genomic DNA was extracted and the V4 region of the 16S rRNA gene was sequenced and analyzed at stage 4 and 5 CKD. Two‐way ANOVA compared severity of CKD (stage 4 vs. 5) and fiber (10% inulin in the diet (gray bar) compared to cellulose (black bar) in the diet). The mean value from the normal animals from both time points are shown as the dashed black line and was not included in the statistical model. The *p* values for age, inulin, and an interaction of age and inulin are shown in each graph. (*A*–*D*) Show the genera that increased after inulin treatment (*Bacteroides*, *Allobaculum*, *Sutterella*, and *Bifidobacterium*), all of them showing a main effect of inulin and only *Sutterella* with an inulin‐by‐age interaction, where inulin‐treatment had a higher relative abundance compared to untreated CKD. (*E*–*H*) Show the genera in which inulin treatment lowered relative abundance (*Lactobacillus*, unclassified Clostridiaceae and Ruminococcaceae, and *Dorea*), all of them showing a main effect of inulin. Data from normal littermates is shown as the dashed black line and was not included in the statistical model. The *p* values for age, inulin, and an interaction of age and inulin are shown in each graph. There was no change from stage 4 to 5 CKD in any of the genera.

Taxonomical analyses at the phyla‐level showed that Firmicutes and Bacteroidetes were the most abundant phyla across all rat groups accounting for over 95% of the relative abundance in all groups at both time points. Phyla relative abundance was similar between NL and CKD, but inulin‐treated rats had higher Bacteroidetes (*p* = 0.03, Fig. 8B,C) and tended to have lower the relative abundance of Firmicutes (*p* = 0.09, Fig. 8D). At the genus‐level, composition was similar between NL and CKD. When comparing CKD with and without inulin, the inulin treatment led to a higher relative abundance of *Bifidobacterium*, *Bacteroides*, *Sutterella*, *Bernesiellaceae*, *Allobaculum*, S24‐7, and unclassified Lachnospiraceae (all *p* < 0.001), and a lower relative abundance of *Lactobacillus*, *Oscillospira*, *Adlercreutzia*, *Dorea*, and unclassified Clostridiaceae, Ruminococcaceae, Rikenellaceae, and Peptostreptococcaceae (all *p* < 0.03, Figs. 8 and S5).

## Discussion

We examined the impact on the administration of the fermentable fiber inulin on CKD‐MBD at two time points, 30 and 32 weeks, equating to stage 4 and 5 CKD in the Cy/+ rat. It is this period of rapid CKD‐MBD progression, where the increased PTH and FGF23 moves from appropriate compensation for decreased renal excretion of phosphate to a state where the persistent increase of PTH and FGF23 can no longer compensate, and end‐organ effects of CKD‐MBD such as bone loss due to cortical porosity, vascular calcification, and LVH ensue in humans,^[^ ^38^ ^]^ as we saw in our rats. In the untreated CKD rats, there was a marked increase in the severity of CKD‐MBD. However, the administration of inulin reduced the rise in phosphorus and PTH, lowered bone remodeling (bone formation rate, osteoclast surface) and prevented cortical changes (porosity, thickness), effectively preventing the dramatic worsening from stage 4 to 5. However, we did not see inulin‐induced alterations in bone mechanics, likely due to the improvement, but not complete amelioration of CKD‐MBD. In contrast to these favorable changes, inulin had insignificant effect on FGF23 levels but there was still a reduction in LVH and aorta/cardiac calcification. Thus, inulin had positive effects, but did not completely reverse the sequelae of CKD‐MBD. Indeed, despite the “reduced” PTH with inulin at stage 5, levels were still over 1000 pg/mL, so it is likely that concomitant therapy with PTH lowering agents such as calcimimetics or vitamin D may be needed to fully suppress PTH and improve bone mechanics.

In the current study, inulin reduced arterial calcification and left ventricular mass index, and trended to reduce cardiac calcification. We have previously shown that this rat model develops concentric LVH with fibrosis (with increased expression of transforming growth factor beta; TGF‐β) and associated arrhythmias.^[^ ^37^ ^]^ It is interesting that we also observed that inulin reduced TGF‐β, indicating that inulin may improve cardiac outcomes in CKD, the leading cause of death. Elevated phosphate levels are traditionally thought of the key mediator of arterial calcification.^[^ ^39^ ^]^ However, in rodents, the oral administration of indoxyl sulfate (above that already elevated in CKD) led to arterial calcification in the adenine rat,^[^ ^40^ ^]^ supporting in vitro studies showing a direct effect this uremic toxin to induce calcification in cultured vascular smooth muscle cells.^[^ ^41^ ^]^ Thus, it is plausible that a reduction in phosphate and a reduction in indoxyl sulfate may be additive to reduce arterial calcification and improve cardiac outcomes. Indeed, studies using the adsorbant AST‐120 also reported improvements in cardiac dysfunction by echocardiography in mice,^[^ ^42^ ^]^ and decreased fibrosis in dogs.^[^ ^43^ ^]^ These studies suggest that intestinal uremic toxin reduction with fiber and/or adsorbents may prove beneficial to cardiac dysfunction in CKD, but much work remains to confirm this exciting possibility.

We hypothesize that these positive effects of the simple dietary intervention of inulin was due to alterations in the gut microbiome. The gut microbiome plays a critical role in uremic toxin accumulation due to increased production of microbially‐derived toxins together with reduced renal clearance.^[^ ^44^, ^45^, ^46^ ^]^ The dysbiosis of CKD is exacerbated by large pill burden including phosphate binders, frequent antibiotic use, and metabolic acidosis.^[^ ^47^ ^]^ We found that inulin altered the cecal microbiota alpha and beta diversity, and induced important taxonomical changes‐ a higher relative abundance of *Allobaculum*, *Bifidobacterium*, *Bacteroides*, and unclassified Lachnospiraceae. This alteration in the gut microbiome also translated to a reduction in gut‐derived uremic toxins and we postulate that effect on indoxyl sulfate was the main driver of the positive effects on bone and vascular calcification. At levels comparable to patients with CKD, in vitro studies of indoxyl sulfate suppress mineralization in rodent osteoblasts,^[^ ^48^ ^]^ downregulate the PTH receptor expression in primary mouse osteoblasts,^[^ ^49^ ^]^ and inhibit receptor activator of nuclear factor κB ligand (RANKL)‐dependent differentiation of osteoclasts.^[^ ^50^ ^]^ In young mice with CKD from 5/6th nephrectomy, indoxyl sulfate worsened bone quantity by μCT.^[^ ^51^ ^]^ Indole fed to rats after a parathyroidectomy worsened the parathyroidectomy induced low bone formation by histomorphometry.^[^ ^52^ ^]^ These results are consistent with our findings of reduced bone formation rate, decreased osteoclast surface, and decreased cortical porosity, although it is not possible to separate out primary effects of inulin from secondary effects from the decrease in PTH.

Fermentable fiber, including inulin, can also alter intraluminal pH, improve mineral solubility, and enhance mineral absorption.^[^ ^11^ ^]^ Interestingly, we did see a reduction of phosphate and PTH with inulin, but not FGF23, which suggests that our results were not predominately due to reduced intestinal phosphate flux. To assess this, we examined intestinal phosphate transporters and found no effect of inulin on expression, albeit we only measured mRNA expression and not protein expression or flux and did not assess colonic transporter expression. Inulin increased FGF23 at stage 4 but had no effect at stage 5 for unclear reasons (although both were extremely elevated levels). We did find increased FGF23 mRNA expression in total bone at stage 4, confirming increased bone production of FGF23 (unpublished observation). It is conceivable that the inulin induced increase in FGF23 at stage 4 led to a reduction in PTH at stage 5 with enhanced urinary phosphate excretion. A previous study in unilateral nephrectomy rats given partially hydrolyzed guar gum (minimally viscous, fermentable fiber) found a decrease in urinary phosphate excretion,^[^ ^53^ ^]^ going against this potential explanation. We did not see a change in serum calcium to account for the changes in PTH, although we measured total calcium. Additional balance studies that assess ionized calcium in the blood and urinary and fecal excretion of both ions are needed to further understand this complex physiology. Unraveling this will require these complicated controlled feeding and formal balance studies in our rat model.

In conclusion, we observed that the administration of inulin to the diet improved parameters of CKD‐MBD in our preclinical model. Although the presumed mechanism was a reduction in gut‐derived uremic toxins via changes in the gut microbiota composition, additional mineral balance studies to rule out changes in intestinal transport in both our rat model and in humans are warranted.

## Author Contributions

**Annabel Biruete:** Conceptualization; formal analysis; investigation; visualization; writing – original draft; writing – review and editing. **Neal Chen:** Conceptualization; formal analysis; investigation; methodology; project administration; supervision; validation; writing – original draft; writing – review and editing. **Corinne E. Metzger:** Investigation; validation; visualization; writing – original draft; writing – review and editing. **Shruthi Srinivasan:** Data curation; investigation; writing – review and editing. **Kalisha O'Neill:** Data curation; formal analysis; investigation; writing – review and editing. **Paul Fallen:** Investigation; writing – review and editing. **Austin Fonseca:** Investigation; writing – review and editing. **Hannah Wilson:** Investigation; writing – review and editing. **Henriette de Loor:** Data curation; investigation; writing – review and editing. **Pieter Evenepoel:** Conceptualization; methodology; project administration; writing – review and editing. **Kelly Swanson:** Formal analysis; investigation; writing – review and editing. **Matt Allen:** Conceptualization; data curation; formal analysis; project administration; supervision; writing – review and editing. **Sharon M Moe:** Conceptualization; funding acquisition; methodology; project administration; resources; supervision; writing – original draft; writing – review and editing.

## Disclosures

There are no related or potential conflicts of interest. Annabel Biruete has received honoraria from Amgen. Sharon M. Moe is a scientific consultant for Sanifit, Inozyme, and Ardelyx who have other products in the field of CKD‐MBD.

### Peer Review

The peer review history for this article is available at https://www.webofscience.com/api/gateway/wos/peer-review/10.1002/jbm4.10837.

## Supporting information


**Data S1.** Supplementary Microbiome Analyses.
**Table S1.** Change in kidney function and body weight.
**Fig. S1.** Bone mechanical properties were reduced in CKD and not improved with inulin treatment.
**Fig. S2.** Intestinal phosphate transporter expression at CKD stage 5.
**Fig. S3.** Other measured metabolites that were not reduced with inulin.
**Fig. S4.** Inulin treatment leads to changes in α‐diversity and β‐diversity.
**Fig. S5.** Additional taxa that were differentially affected by inulin.

## Data Availability

Data is available from Dr. Moe upon request.

## References

[1] Moe SM , Drueke T , Lameire N , Eknoyan G . Chronic kidney disease‐mineral‐bone disorder: a new paradigm. Adv Chronic Kidney Dis. 2007;14(1):3–12. 17200038 10.1053/j.ackd.2006.10.005

[2] Moe SM , Nickolas TL . Fractures in patients with CKD: time for action. Clin J Am Soc Nephrol. 2016;11(11):1929–1931. 27797903 10.2215/CJN.09500916 PMC5108207

[3] Coco M , Rush H . Increased incidence of hip fractures in dialysis patients with low serum parathyroid hormone. Am J Kidney Dis. 2000;36(6):1115–1121. 11096034 10.1053/ajkd.2000.19812

[4] Fried LF , Biggs ML , Shlipak MG , et al. Association of kidney function with incident hip fracture in older adults. J Am Soc Nephrol. 2007;18(1):282–286. 17167115 10.1681/ASN.2006050546

[5] Cheung AK , Sarnak MJ , Yan G , et al. Atherosclerotic cardiovascular disease risks in chronic hemodialysis patients. Kidney Int. 2000;58(1):353–362. 10886582 10.1046/j.1523-1755.2000.00173.x

[6] Evenepoel P , Dejongh S , Verbeke K , Meijers B . The role of gut dysbiosis in the bone‐vascular Axis in chronic kidney disease. Toxins (Basel). 2020;12(5):285. 32365480 10.3390/toxins12050285 PMC7290823

[7] Block GA , Hulbert‐Shearon TE , Levin NW , Port FK . Association of serum phosphorus and calcium x phosphate product with mortality risk in chronic hemodialysis patients: a national study. Am J Kidney Dis. 1998;31(4):607–617. 9531176 10.1053/ajkd.1998.v31.pm9531176

[8] Floege J , Kim J , Ireland E , et al. Serum iPTH, calcium and phosphate, and the risk of mortality in a European haemodialysis population. Nephrol Dial Transplant. 2011;26(6):1948–1955. 20466670 10.1093/ndt/gfq219 PMC3107766

[9] Massry SG , Smogorzewski M . Mechanisms through which parathyroid hormone mediates its deleterious effects on organ function in uremia. Semin Nephrol. 1994;14(3):219–231. 8036356

[10] Faul C , Amaral AP , Oskouei B , et al. FGF23 induces left ventricular hypertrophy. J Clin Invest. 2011;121(11):4393–4408. 21985788 10.1172/JCI46122 PMC3204831

[11] Hughes RL , Alvarado DA , Swanson KS , Holscher HD . The prebiotic potential of inulin‐type fructans: a systematic review. Adv Nutr. 2021;13:492–529. 10.1093/advances/nmab119 PMC8970830 34555168

[12] Biruete A , Cross TL , Allen JM , et al. Effect of dietary inulin supplementation on the gut microbiota composition and derived metabolites of individuals undergoing hemodialysis: a pilot study. J Ren Nutr. 2021;31(5):512–522. 34120835 10.1053/j.jrn.2020.10.003 PMC8403151

[13] Camerotto C , Cupisti A , D'Alessandro C , Muzio F , Gallieni M . Dietary fiber and gut microbiota in renal diets. Nutrients. 2019;11(9):2149. 31505733 10.3390/nu11092149 PMC6770883

[14] Tawfeek H , Bedi B , Li JY , et al. Disruption of PTH receptor 1 in T cells protects against PTH‐induced bone loss. PLoS One. 2010;5(8):e12290. 20808842 10.1371/journal.pone.0012290 PMC2924900

[15] Yu M , Malik Tyagi A , Li JY , et al. PTH induces bone loss via microbial‐dependent expansion of intestinal TNF(+) T cells and Th17 cells. Nat Commun. 2020;11(1):468. 31980603 10.1038/s41467-019-14148-4 PMC6981196

[16] Di Iorio BR , Rocchetti MT , De Angelis M , et al. Nutritional therapy modulates intestinal microbiota and reduces serum levels of total and free indoxyl sulfate and P‐Cresyl sulfate in chronic kidney disease (Medika study). J Clin Med. 2019;8(9):1424. 31510015 10.3390/jcm8091424 PMC6780815

[17] Dai Z , Zhang Y , Lu N , Felson DT , Kiel DP , Sahni S . Association between dietary fiber intake and bone loss in the Framingham offspring study. J Bone Miner Res. 2018;33(2):241–249. 29024045 10.1002/jbmr.3308 PMC5990003

[18] Liu Z , Chen B , Li B , et al. Greater consumption of total and individual lignans and dietary fibers were significantly associated with lowered risk of hip fracture‐a 1:1 matched case‐control study among Chinese elderly men and women. Nutrients. 2022;14(5):1100. 35268074 10.3390/nu14051100 PMC8912333

[19] Threapleton DE , Greenwood DC , Evans CE , et al. Dietary fibre intake and risk of cardiovascular disease: systematic review and meta‐analysis. BMJ. 2013;347:f6879. 24355537 10.1136/bmj.f6879 PMC3898422

[20] Hoff S , Halbritter J , Epting D , et al. ANKS6 is a central component of a nephronophthisis module linking NEK8 to INVS and NPHP3. Nat Genet. 2013;45(8):951–956. 23793029 10.1038/ng.2681 PMC3786259

[21] Bakey Z , Bihoreau MT , Piedagnel R , et al. The SAM domain of ANKS6 has different interacting partners and mutations can induce different cystic phenotypes. Kidney Int. 2015;88(2):299–310. 26039630 10.1038/ki.2015.122

[22] Cowley BD Jr , Gudapaty S , Kraybill AL , et al. Autosomal‐dominant polycystic kidney disease in the rat. Kidney Int. 1993;43(3):522–534. 8455352 10.1038/ki.1993.79

[23] Vorland CJ , Lachcik PJ , Swallow EA , et al. Effect of ovariectomy on the progression of chronic kidney disease‐mineral bone disorder (CKD‐MBD) in female Cy/+ rats. Sci Rep. 2019;9(1):7936. 31138895 10.1038/s41598-019-44415-9 PMC6538713

[24] Isakova T , Wolf MS . FGF23 or PTH: which comes first in CKD? Kidney Int. 2010;78(10):947–949. 21030968 10.1038/ki.2010.281

[25] Moe SM , Chen NX , Newman CL , et al. Anti‐sclerostin antibody treatment in a rat model of progressive renal osteodystrophy. J Bone Miner Res. 2015;30(3):499–509. 25407607 10.1002/jbmr.2372 PMC4333005

[26] Avin KG , Allen MR , Chen NX , et al. Voluntary wheel running has beneficial effects in a rat model of CKD‐mineral bone disorder (CKD‐MBD). J Am Soc Nephrol. 2019;30(10):1898–1909. 31501355 10.1681/ASN.2019040349 PMC6779348

[27] de Loor H , Poesen R , De Leger W , et al. A liquid chromatography ‐ tandem mass spectrometry method to measure a selected panel of uremic retention solutes derived from endogenous and colonic microbial metabolism. Anal Chim Acta. 2016;936:149–156. 27566350 10.1016/j.aca.2016.06.057

[28] Guo J , Liu M , Yang D , et al. Suppression of Wnt signaling by Dkk1 attenuates PTH‐mediated stromal cell response and new bone formation. Cell Metab. 2010;11(2):161–171. 20142103 10.1016/j.cmet.2009.12.007 PMC2819982

[29] Dempster DW , Compston JE , Drezner MK , et al. Standardized nomenclature, symbols, and units for bone histomorphometry: a 2012 update of the report of the ASBMR Histomorphometry Nomenclature Committee. J Bone Miner Res. 2013;28(1):2–17. 23197339 10.1002/jbmr.1805 PMC3672237

[30] Hirano T , Turner CH , Forwood MR , Johnston CC , Burr DB . Does suppression of bone turnover impair mechanical properties by allowing microdamage accumulation? Bone. 2000;27(1):13–20. 10865204 10.1016/s8756-3282(00)00284-2

[31] Chen NX , O'Neill KD , Allen MR , Newman CL , Moe SM . Low bone turnover in chronic kidney disease is associated with decreased VEGF‐A expression and osteoblast differentiation. Am J Nephrol. 2015;41(6):464–473. 26228644 10.1159/000438461

[32] Chen NX , Srinivasan S , O'Neill K , et al. Effect of advanced glycation end‐products (AGE) lowering drug ALT‐711 on biochemical, vascular, and bone parameters in a rat model of CKD‐MBD. J Bone Miner Res. 2020;35(3):608–617. 31743501 10.1002/jbmr.3925 PMC9030558

[33] Moe SM , Duan D , Doehle BP , O'Neill KD , Chen NX . Uremia induces the osteoblast differentiation factor Cbfa1 in human blood vessels. Kidney Int. 2003;63(3):1003–1011. 12631081 10.1046/j.1523-1755.2003.00820.x

[34] Biruete A , Srinivasan S , O'Neill KD , et al. Adverse effects of autoclaved diets on the progression of chronic kidney disease (CKD) and CKD‐mineral and bone disorder in rats. Am J Nephrology. 2020;51:381–389. 10.1159/000506729 PMC7228841 32146472

[35] Biruete A , Metzger CE , Chen NX , et al. Effects of ferric citrate and intravenous iron sucrose on markers of mineral, bone, and iron homeostasis in a rat model of CKD‐MBD. Nephrol Dial Transplant. 2022;37(10):1857–1867. 35482713 10.1093/ndt/gfac162 PMC9494145

[36] Moe SM , Chen NX , Newman CL , et al. A comparison of calcium to zoledronic acid for improvement of cortical bone in an animal model of CKD. J Bone Miner Res. 2014;29(4):902–910. 24038306 10.1002/jbmr.2089 PMC3940692

[37] Hsueh CH , Chen NX , Lin SF , et al. Pathogenesis of arrhythmias in a model of CKD. J Am Soc Nephrol. 2014;25(12):2812–2821. 24854269 10.1681/ASN.2013121343 PMC4243359

[38] Isakova T , Wahl P , Vargas GS , et al. Fibroblast growth factor 23 is elevated before parathyroid hormone and phosphate in chronic kidney disease. Kidney Int. 2011;79(12):1370–1378. 21389978 10.1038/ki.2011.47 PMC3134393

[39] Moe SM , Chen NX . Pathophysiology of vascular calcification in chronic kidney disease. Circ Res. 2004;95(6):560–567. 15375022 10.1161/01.RES.0000141775.67189.98

[40] Opdebeeck B , Maudsley S , Azmi A , et al. Indoxyl sulfate and p‐Cresyl sulfate promote vascular calcification and associate with glucose intolerance. J Am Soc Nephrol. 2019;30(5):751–766. 30940651 10.1681/ASN.2018060609 PMC6493976

[41] He X , Jiang H , Gao F , Liang S , Wei M , Chen L . Indoxyl sulfate‐induced calcification of vascular smooth muscle cells via the PI3K/Akt/NF‐kappaB signaling pathway. Microsc Res Tech. 2019;82(12):2000–2006. 31448474 10.1002/jemt.23369

[42] Shen WC , Chou YH , Shi LS , et al. AST‐120 improves cardiac dysfunction in acute kidney injury mice via suppression of apoptosis and proinflammatory NF‐kappaB/ICAM‐1 signaling. J Inflamm Res. 2021;14:505–518. 33658826 10.2147/JIR.S283378 PMC7917393

[43] Asanuma H , Chung H , Ito S , et al. AST‐120, an adsorbent of uremic toxins, improves the pathophysiology of heart failure in conscious dogs. Cardiovasc Drugs Ther. 2019;33(3):277–286. 30903544 10.1007/s10557-019-06875-z

[44] Duranton F , Cohen G , De Smet R , et al. Normal and pathologic concentrations of uremic toxins. J Am Soc Nephrol. 2012;23(7):1258–1270. 22626821 10.1681/ASN.2011121175 PMC3380651

[45] Vanholder R , Glorieux G , Lameire N . Uraemic toxins and cardiovascular disease. Nephrol Dial Transplant. 2003;18(3):463–466. 12584262 10.1093/ndt/18.3.463

[46] Gryp T , De Paepe K , Vanholder R , et al. Gut microbiota generation of protein‐bound uremic toxins and related metabolites is not altered at different stages of chronic kidney disease. Kidney Int. 2020;97(6):1230–1242. 32317112 10.1016/j.kint.2020.01.028

[47] Biruete A , Hill Gallant KM , Lindemann SR , Wiese GN , Chen NX , Moe SM . Phosphate binders and nonphosphate effects in the gastrointestinal tract. J Ren Nutr. 2020;30(1):4–10. 30846238 10.1053/j.jrn.2019.01.004 PMC6722023

[48] Watanabe H , Sugimoto R , Ikegami K , et al. Parathyroid hormone contributes to the down‐regulation of cytochrome P450 3A through the cAMP/PI3K/PKC/PKA/NF‐kappaB signaling pathway in secondary hyperparathyroidism. Biochem Pharmacol. 2017;145:192–201. 28843775 10.1016/j.bcp.2017.08.016

[49] Nii‐Kono T , Iwasaki Y , Uchida M , et al. Indoxyl sulfate induces skeletal resistance to parathyroid hormone in cultured osteoblastic cells. Kidney Int. 2007;71(8):738–743. 17264878 10.1038/sj.ki.5002097

[50] Mozar A , Louvet L , Godin C , et al. Indoxyl sulphate inhibits osteoclast differentiation and function. Nephrol Dial Transplant. 2012;27(6):2176–2181. 22140126 10.1093/ndt/gfr647

[51] Liu WC , Shyu JF , Lim PS , et al. Concentration and duration of indoxyl sulfate exposure affects Osteoclastogenesis by regulating NFATc1 via aryl hydrocarbon receptor. Int J Mol Sci. 2020;21(10):3486. 32429048 10.3390/ijms21103486 PMC7278944

[52] Hirata J , Hirai K , Asai H , et al. Indoxyl sulfate exacerbates low bone turnover induced by parathyroidectomy in young adult rats. Bone. 2015;79:252–258. 26112820 10.1016/j.bone.2015.06.010

[53] Tani M , Tanaka S , Takamiya K , et al. Effects of dietary fiber on vascular calcification by repetitive diet‐induced fluctuations in plasma phosphorus in early‐stage chronic kidney disease rats. J Clin Biochem Nutr. 2020;67(3):283–289. 33293769 10.3164/jcbn.20-46 PMC7705083

