# Supplementary material for: The Dietary Fiber Inulin Slows Progression of Chronic Kidney Disease–Mineral Bone Disorder (CKD‐MBD) in a Rat Model of CKD
Source: JBMR Plus. 2023 Dec 7;7(12):e10837. doi: 10.1002/jbm4.10837 (PMC10731114; doi:10.1002/jbm4.10837)
Supplement: Supplementary file 1 — Data S1. Supplementary Microbiome Analyses. Table S1. Change in kidney function and body weight. Fig. S1. Bone mechanical properties were reduced in CKD and not improved with inulin treatment. Fig. S2. Intestinal phosphate transporter expression at CKD stage 5. Fig. S3. Other measured metabolites that were not reduced with inulin. Fig. S4. Inulin treatment leads to changes in α‐diversity and β‐diversity. Fig. S5. Additional taxa that were differentially affected by inulin. [file JBM4-7-e10837-s001.docx]

**SUPPLEMENT Table of Content:**

**Supplementary Microbiome Analyses:**

**Supplemental Table 1**: Change in kidney function and body weight

**Supplemental Figure 1:** Bone mechanical properties were reduced in CKD and not improved with inulin treatment

**Supplemental Figure 2:** Intestinal phosphate transporter expression at CKD stage 5

**Supplemental Figure 3**: Other measured metabolites that were not reduced with inulin

**Supplemental Figure 4:** Inulin treatment leads to changes in α-diversity and β-diversity

**Supplemental Figure 5:** Additional taxa that were differentially affected by inulin (

**Supplementary Methods to Assess microbiome:**

Total genomic DNA from cecal digesta (proximal large intestine) were extracted (Qiagen, Ann Arbor, MI), double-stranded DNA was quantified using the Clariostar spectrometer (BMG Labtech, Cary NC), and quality assessed by electrophoresis with 2% Agarose EX-gels using the E-Gel iBase (Invitrogen, Grand Island, NY). Fluidigm Access Array was used to generate 16S rRNA gene amplicons, in combination with Roche High Fidelity Fast Start Kit. Primers 515 F and 806R targeting a 252 bp-fragment of the V4 region of the bacterial 16S rRNA were amplified [1]. Detailed methods are provided in the supplementary material. CS1 forward tag and CS2 reverse tag were added according to the Fluidigm protocol. Sequencing was performed through Illumina Mi-seq using V3 reagents and performed at the University of Illinois. Relative changes in bacterial diversity (α-diversity and β-diversity) and taxonomical changes were analyzed through the open software QIIME2 (version 2019.10[2, 3]). In short, forward reads were imported using the single-end Earth Microbiome Project (EMP) protocol and demultiplexed using the demux plugin. Sequences were then denoised using DADA-2[4]. For diversity analyses, samples were rarified to a sampling depth of 29,050. Amplicon sequence variants (ASVs) were classified using the GreenGenes database version 13_8[5]. A phylogenetic tree was created using the align-to-tree-mafft-fasttree pipeline. The α- and β-diversity metrics were computed using the q2-diversity plugin (core-metrics-phylogenetic) using rarified counts. For α-diversity (number of ASVs, Shannon diversity index, and Faith’s phylogenetic diversity), we tested the difference between CKD with and without inulin at stages 4 and 5 via 2-way ANOVA, representing the mean of normal rats visually in graphs. For β-diversity, we tested the difference between normal, CKD, and CKD with inulin at stages 4 and 5 using permutational multivariate analysis of variance (PERMANOVA) on unweighted and weighted UniFrac distance matrices and were visualized using EMPEROR.

1. Caporaso JG, Lauber CL, Walters WA*, et al.* Global patterns of 16S rRNA diversity at a depth of millions of sequences per sample. Proc Natl Acad Sci U S A 2011;108 Suppl 1:4516-4522

2. Bolyen E, Rideout JR, Dillon MR*, et al.* Author Correction: Reproducible, interactive, scalable and extensible microbiome data science using QIIME 2. Nat Biotechnol 2019;37(9):1091

3. Bolyen E, Rideout JR, Dillon MR*, et al.* Reproducible, interactive, scalable and extensible microbiome data science using QIIME 2. Nat Biotechnol 2019;37(8):852-857

4. Callahan BJ, McMurdie PJ, Rosen MJ*, et al.* DADA2: High-resolution sample inference from Illumina amplicon data. Nat Methods 2016;13(7):581-583

5. DeSantis TZ, Hugenholtz P, Larsen N*, et al.* Greengenes, a chimera-checked 16S rRNA gene database and workbench compatible with ARB. Appl Environ Microbiol 2006;72(7):5069-5072

**Supplemental Table 1: Change in kidney function and body weight**

| **Treatment groups** | **Body weight (g)** | | **Kidney weight (g)** | | **BUN (mg/dL)** | | **Creatinine (mg/dL)** | |
| --- | --- | --- | --- | --- | --- | --- | --- | --- |
|  | Stage 4 | Stage 5 | Stage 4 | Stage 5 | Stage 4 | Stage 5 | Stage 4 | Stage 5 |
| Normal | 525 ±36 | 524±19 | 3.44±0.38 | 3.45±0.16 | 18.21±2.41 | 17.35±3.34 | 0.59±0.13 | 0.63±0.08 |
| CKD | 503±35 | 453±77* | 5.72±0.71* | 7.44±1.46* | 43.60±5.86* | 46.15±10.12* | 1.27±0.29* | 1.96±0.81* |
| CKD/  10% inulin | 508±33 | 488±42* | 6.24±1.41* | 6.35±0.88*# | 45.13±7.59* | 42.18±5.33* | 1.22±0.25* | 1.73±0.41* |

Data are shown as mean ± SD (n =10-12 rats each group). *p < 0.05, NL vs. CKD; #p<0.05, CKD vs. CKD/10% inulin by ANOVA at each stage (Stage 4 and stage 5).

**Supplemental Figure 1: Bone mechanical properties were reduced in CKD and not improved with inulin** **treatment**
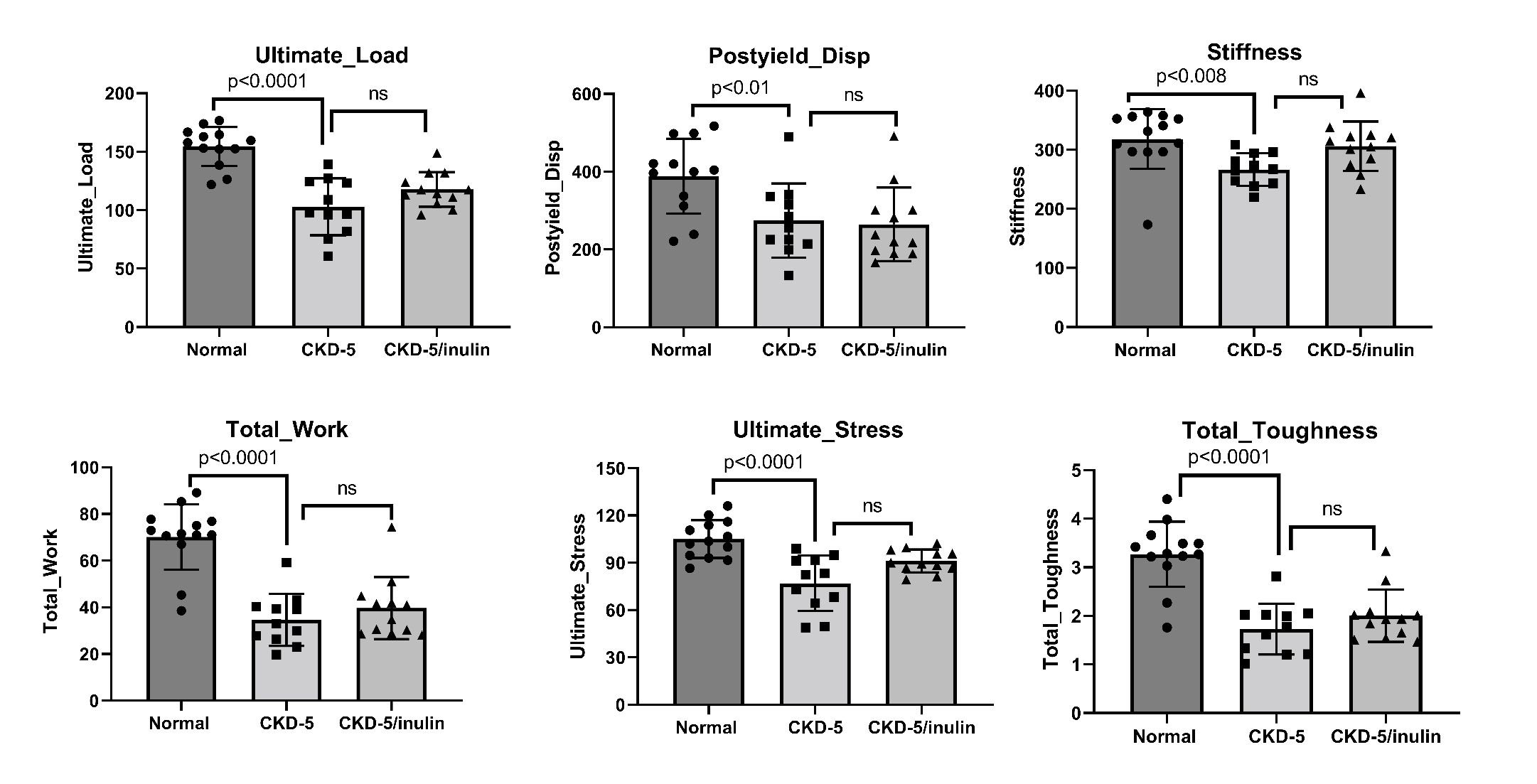
**Supplemental Figure 1**: Bone was collected at the time of euthanasia at CKD stage 5 and analyzed by 4-point bending. Three groups were compared by ANOVA with post hoc testing: Normal animals (black circle symbols, dark gray), CKD fed cellulose diet (black square symbols, light gray bar) and CKD animals fed inulin (black triangle symbols, medium gray bar). As shown in all of the panels, CKD bone quality was significantly worse to NL animals by all measures, but there was no improvement seen when the animals were fed inulin. N = 10-12 for each group shown as individual symbols.

**Supplemental Figure 2: Intestinal expression of phosphate transporters at CKD stage 5:**


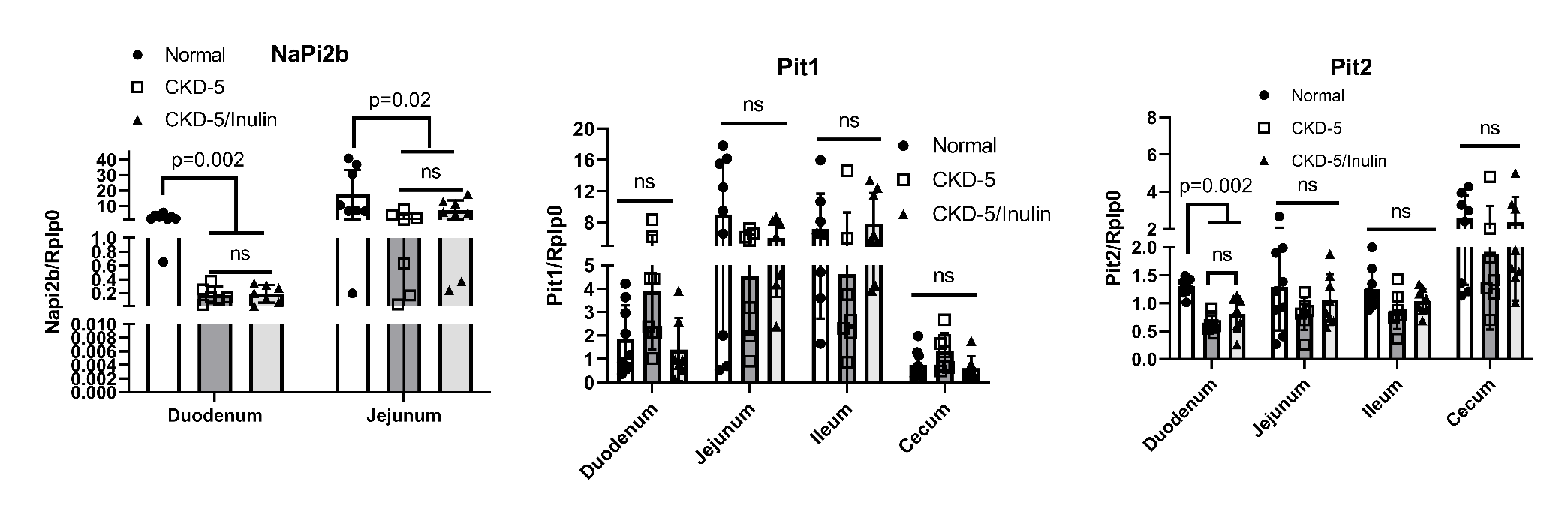


**Supplemental Figure 2: Intestinal expression of phosphate transporters at CKD stage 5:** Each intestinal segment was examined by real time PCR for the expression of phosphate transporters and the groups compared by ANOVA. Each graph shows individual transporter expression for different segments for normal animals (black dot symbols/white bars), CKD-5 animals (square symbols/dark gray bars), and CKD-5 animals + inulin (triangle symbols/light gray bars). NaPi2b expression was reduced in CKD-5 animals compared to NL in the duodenum and jejunum, but not affected by inulin and not expressed in the remaining segments. Pit1 was not different in any of the segments. Pit2 was reduced in CKD-5 animals compared to NL in the duodenum but not affected by inulin. N = 8 to 10 per group.

**Supplemental Figure 3**: Other measured metabolites that were not reduced with inulin (additive to Figure 1)


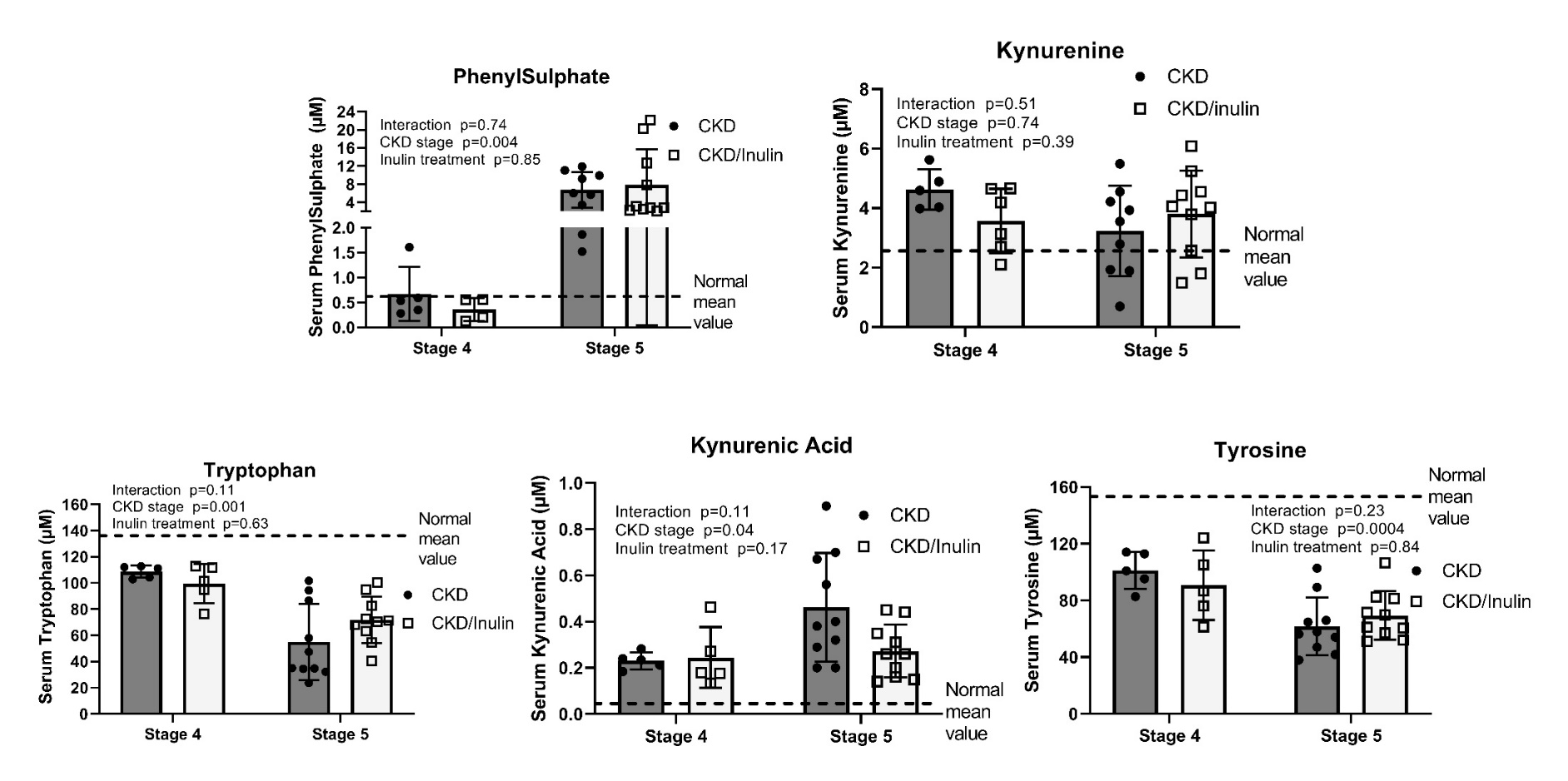


**Supplemental Figure 3:** **Uremic toxins that were not reduced by inulin:** Serum was collected at the time of euthanasia and analyzed by mass spectroscopy. Two-way ANOVA compared severity of CKD (stage 4 vs. stage 5) and diet (10% inulin in the diet (white bar) compared to cellulose (black bar) in the diet). The mean value from the normal animals from both time points are shown as the dashed black line and was not included in the statistical model. The p values for age, inulin, and an interaction of age and inulin are shown in each graph. Except for kynurenine, levels changed with increased age, but inulin had no effect. The p values for age, inulin, and an interaction of age and inulin are shown in each graph. N = 5 -10 for each group.**Supplemental Figure 4: Inulin treatment leads to changes in α-diversity** **and β-diversity.**

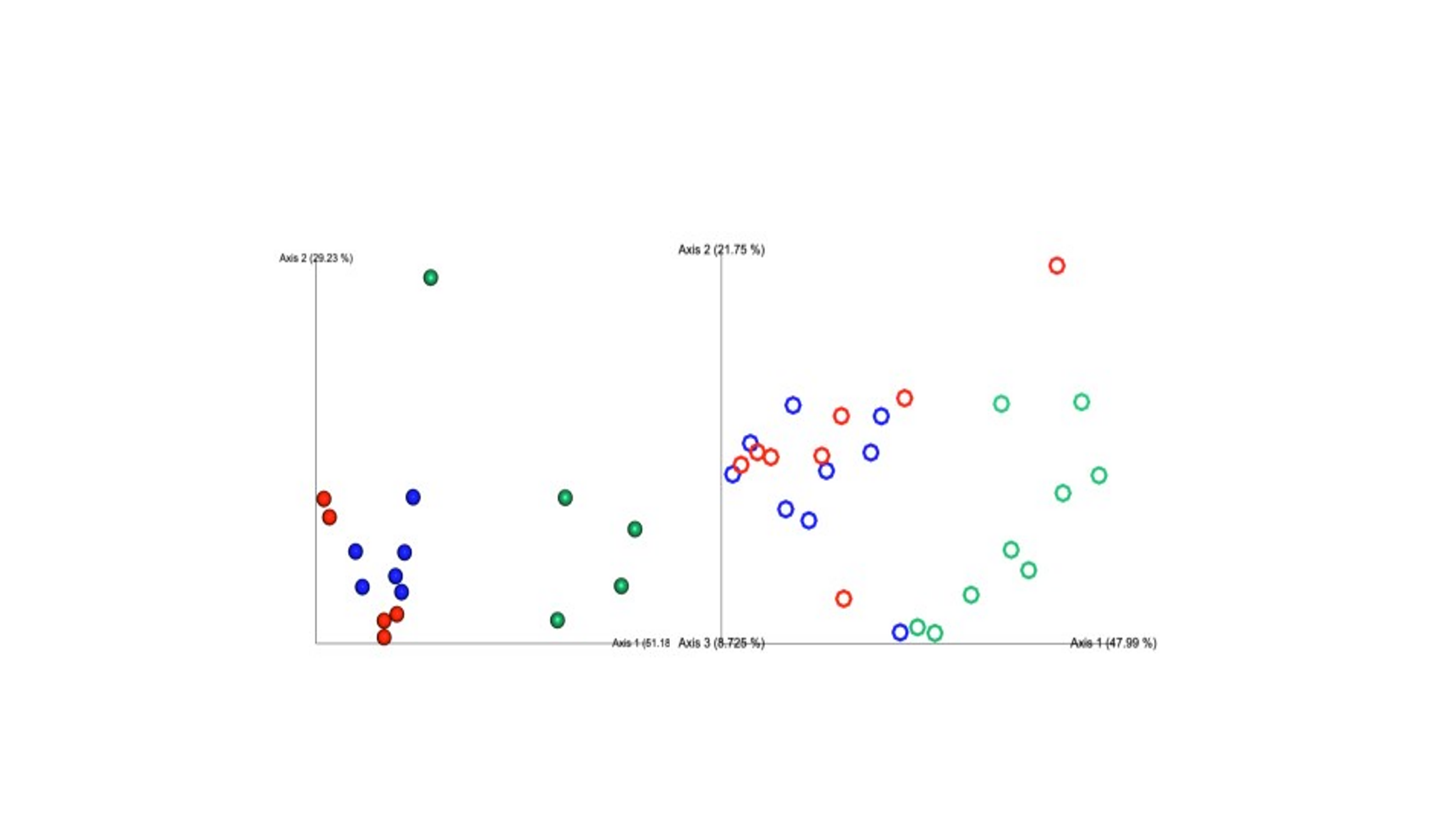


**Supplemental Figure 4:** Inulin treatment leads to changes in α-diversity and β-diversity. Two-way ANOVA compared severity of CKD severity (stage 4 and stage 5) and fiber (10% inulin in the diet (white bar) compared to cellulose (black bar) in the diet). The mean value from the normal animals from both time points are shown as the dashed black line and was not included in the statistical model. Top left: Amplicon sequence variants (ASVs) were classified using the GreenGenes database version 13_8. Top right: Faith’s phylogenetic diversity. The plot below shows the weighted UniFrac distances at stage 4 (closed circles, NL blue, CKD red, CKD/inulin green) and at stage 5 (open circles NL blue, CKD red, CKD/inulin green) where the overall microbial composition was similar between NL and CKD rats at stage 4 and stage 5 (PERMANOVA q>0.05), but inulin-treated rats were different (PERMANOVA q<0.05).

**Supplemental Figure 5: Additional taxa differentially affected by inulin.**


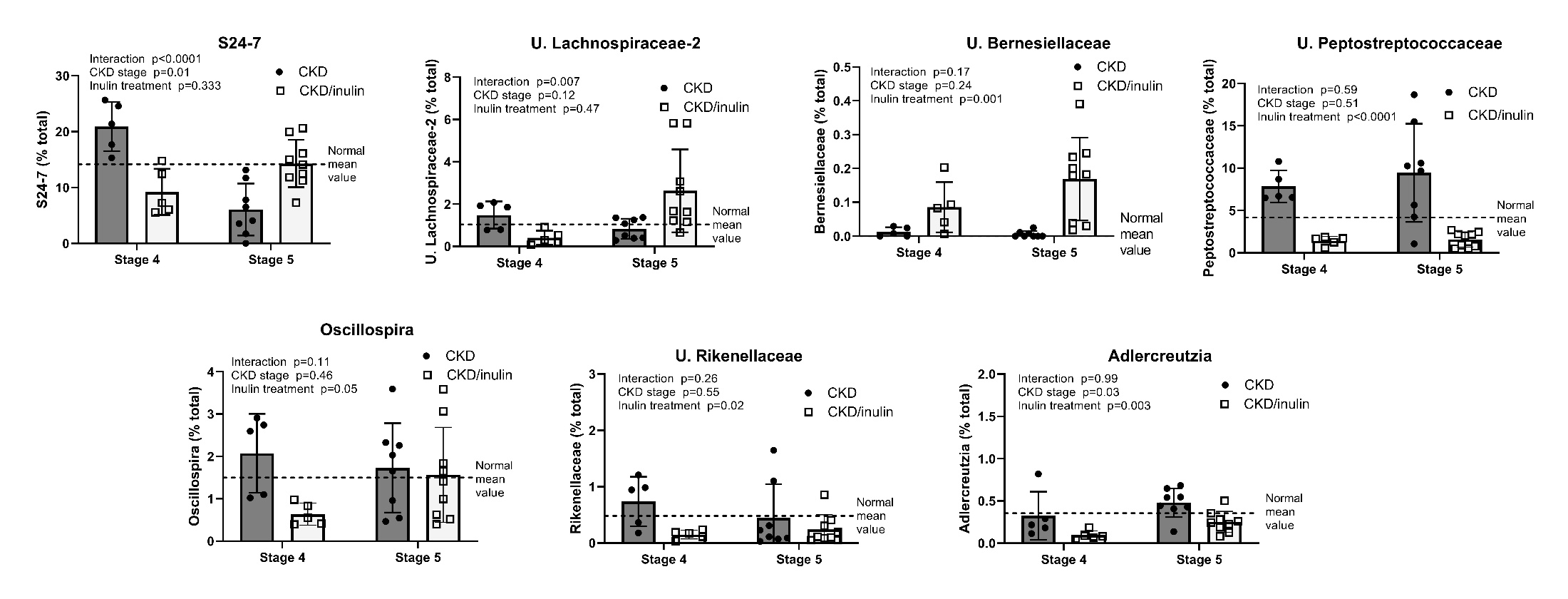
**Supplemental Figure 5:** Two-way ANOVA compared severity of CKD (stage 4 vs. stage 5) and fiber (10% inulin in the diet (white bar) compared to cellulose (black bar) in the diet). The mean value from the normal animals from both time points are shown as the dashed black line and was not included in the statistical model. Inulin led to initial lower relative abundance of S24-7 and unclassified Lachnospiraceae at stage 4, but the abundance increased at stage 5. Unclassified Bernesiellaceae had a higher relative abundance at stage 4 and stage 5 with inulin. Unclassified Peptostretococcaceae had a lower relative abundance with inulin at stage 4 and stage 5. Oscillospira had an initial lower relative abundance with inulin at stage 4, but similar to untreated CKD rats at stage 5. Unclassified Rikenellaceae and Adlercreutzia had a lower relative abundance with inulin treatment at both time points.
